# Boosting the down-shifting luminescence of rare-earth nanocrystals for biological imaging beyond 1500 nm

**DOI:** 10.1038/s41467-017-00917-6

**Published:** 2017-09-29

**Authors:** Yeteng Zhong, Zhuoran Ma, Shoujun Zhu, Jingying Yue, Mingxi Zhang, Alexander L. Antaris, Jie Yuan, Ran Cui, Hao Wan, Ying Zhou, Weizhi Wang, Ngan F. Huang, Jian Luo, Zhiyuan Hu, Hongjie Dai

**Affiliations:** 1 CAS Key Laboratory of Standardization and Measurement for Nanotechnology, CAS Key Laboratory for Biomedical Effects of Nanomaterials and Nanosafety, CAS Center for Excellence in Nanoscience, National Center for Nanoscience and Technology of China, Beijing, 100190 China; 2 Department of Chemistry, Stanford University, Stanford, CA 94305 USA; 3 Stanford Cardiovascular Institute, Stanford University, Stanford, CA 94305 USA; 4 Department of Neurology and Neurological Sciences, School of Medicine, Stanford University, Stanford, CA 94305 USA; 5 Sino-Danish College, University of Chinese Academy of Sciences, Beijing, 100049 China; 6 Yangtze River Delta Academy of Nanotechnology and Industry Development Research, Jiaxing, Zhejiang 314000 China

## Abstract

In vivo fluorescence imaging in the near-infrared region between 1500–1700 nm (NIR-IIb window) affords high spatial resolution, deep-tissue penetration, and diminished auto-fluorescence due to the suppressed scattering of long-wavelength photons and large fluorophore Stokes shifts. However, very few NIR-IIb fluorescent probes exist currently. Here, we report the synthesis of a down-conversion luminescent rare-earth nanocrystal with cerium doping (Er/Ce co-doped NaYbF_4_ nanocrystal core with an inert NaYF_4_ shell). Ce doping is found to suppress the up-conversion pathway while boosting down-conversion by ~9-fold to produce bright 1550 nm luminescence under 980 nm excitation. Optimization of the inert shell coating surrounding the core and hydrophilic surface functionalization minimize the luminescence quenching effect by water. The resulting biocompatible, bright 1550 nm emitting nanoparticles enable fast in vivo imaging of blood vasculature in the mouse brain and hindlimb in the NIR-IIb window with short exposure time of 20 ms for rare-earth based probes.

## Abstract (web summary)

Fluorescence imaging in the near-infrared window between 1500–1700 nm (NIR-IIb window) offers superior spatial resolution and tissue penetration depth, but few NIR-IIb probes exist. Here, the authors synthesize rare earth down-converting nanocrystals as promising fluorescent probes for *in vivo* imaging in this spectral region.

## Introduction

In vivo fluorescence-based optical imaging provides high spatial and temporal resolution, giving researchers the unique ability to visualize biological processes in real-time (30 frames-per-second) down to the cellular level^1–3^. For decades, one-photon fluorescence imaging in the visible (400–700 nm) and traditional near-infrared (NIR-I; 750–900 nm) regions of the electromagnetic spectrum have been plagued by the inability to clearly resolve deep-tissue structures and physiological dynamics^4^. As a recent development, NIR-emissive fluorescent probes in the second near-infrared window (NIR-II, 1000–1700 nm) have afforded improved in vivo fluorescence imaging quality owing to suppressed scattering of photons and diminished auto-fluorescence^5–7^. Several classes of fluorescent NIR-II probes have been reported including carbon nanotubes^6^, conjugated polymers^8^, small molecular dyes^9^ and inorganic-based nanoparticles of quantum dots^7, 10^, and rare-earth nanocrystals^11^. Indeed, progress have been made in NIR-II in vivo biological imaging owing to the development of various NIR-II fluorescent probes^2, 12–14^. Still, bright probes with emission in the long end of the NIR-II region remain scarce and are desired in order to further reduce scatting of emitted photons and maximize in vivo fluorescence imaging depth and clarity.

Recent progress has exemplified the enhanced resolution of vasculature structures in the mouse brain and hindlimb by detecting fluorescence emission in the NIR-IIb window (1500–1700 nm)^10, 15^. The ~ 1600 nm spectral region resides in a local valley of water’s absorption spectrum where the minimal photon absorbance in-between water’s 1st and 2nd overtones enables deep-tissue optical access. In addition, a near zero-background achieved with NIR-IIb probes such as carbon nanotubes eliminates tissue auto-fluorescence by 808 nm excitation in the NIR-I region while detecting fluorescence emission in the >1500 nm NIR-IIb window^16^. Since photon scattering scales as *λ* ^−*α*^ (where *λ* is the wavelength and *α* = 0.2–4 for different tissues), the NIR-IIb window provides the lowest photon scattering in the NIR-II region detectable with a 2D InGaAs camera, offering the most desirable imaging clarity and deep penetration using existing detectors.

Currently NIR-IIb fluorescent probes emitting in the ~1600 nm are still very limited. Previously synthesized semiconducting single-walled nanotubes (SWNTs)^15^ and CdSe@CdS coated InAs quantum dots (QDs)^10^ emitting in the NIR-IIb region developed for imaging mouse brain vasculature require considerably long exposure times (200–5000 ms) due to their low quantum yield (QY) in aqueous biological environments. Other candidates such as PbS^17^ and PbSe^18^ QDs are promising but need to resolve issues of photo-instability in aqueous solutions. As an alternative, erbium doped rare-earth nanoparticles (Er-RENPs) show useful down-conversion (DC) luminescence in NIR-IIb region^11^.

Er-RENP probes, well known for their up-conversion (UC) luminescence, have garnered recent interest for biological imaging applications owing to their low toxicity, narrow band emission, and superior photo/chemical stability^19–21^. Recently, the NIR-IIb DC emission (1550 nm) of Er-based RENPs (Yb/Er co-doped NaYF_4_ nanocrystals) were employed for in vivo NIR-II imaging^11^. A caveat was that a relatively low QY of the RENP in toluene was reported, which would be further quenched after transferring to aqueous solutions due to the strong energy-transfer rate from Er^3+^ ions to the OH^−^ groups in solution^22^. Thus far, it has been challenging to boost the Er-RENPs DC luminescence^23–25^, requiring very long exposure times up to 1 s for in vivo imaging^25^.

A typical Er-RENP is comprised of an Er doped NaYbF_4_ crystalline core containing Er^3+^ ions and Yb^3+^ ions surrounded by an inert shell of NaYF_4_. The Yb^3+^ ions absorb ~ 980 nm light efficiently and can transfer their energy to excite the Er^3+^ ions to the Er ^4^I_11/2_ level. The excited Er ^4^I_11/2_ state can relax non-radiatively to the ^4^I_13/2_ level and then radiatively to the ^4^I_15/2_ level to produce the 1550 nm DC emission. However, two competing processes exist to the down-conversion luminescence. The first is the well-known UC emission process through simultaneous two-photon absorption that excites the ^4^I_11/2_ level to higher levels for subsequent UC emission. The second is quenching of the excited ^4^I_13/2_ state caused by the OH^−^ group when RENP is transferred to an aqueous solution^22^. These competing processes pose challenges to obtaining highly bright NIR-IIb DC emission of Er-RENPs for biological imaging.

Here, we report an Er-based RENP with a 2% Er and 2% Ce doped NaYbF_4_ core and NaYF_4_ shell (named NaYbF_4_:2%Er,2%Ce@NaYF_4_) with high QY of DC emission at ~ 1550 nm under a 980 nm excitation for NIR-IIb imaging. The down-conversion 1550 nm emission is boosted by ~ 9-fold with the UC pathway highly suppressed through a key approach of Ce doping in the core, while the aqueous quenching effect was alleviated by optimizing of the thickness of the NaYF_4_ inert shell. The resulting RENPs exhibit the brightest 1550 nm emission in aqueous solutions under 980 nm excitation among rare-earth DC nanoparticles thus far. These bright RENPs enable non-invasive through-skull/scalp mouse brain imaging (980 nm excitation/1500–1700 nm detection) in the NIR-IIb window using short exposure times (20 ms) for rare-earth luminescent probes.

## Results

### Ce doping enhanced down-conversion for NIR-IIb emission of Er-RENPs

**Fig. 1:**
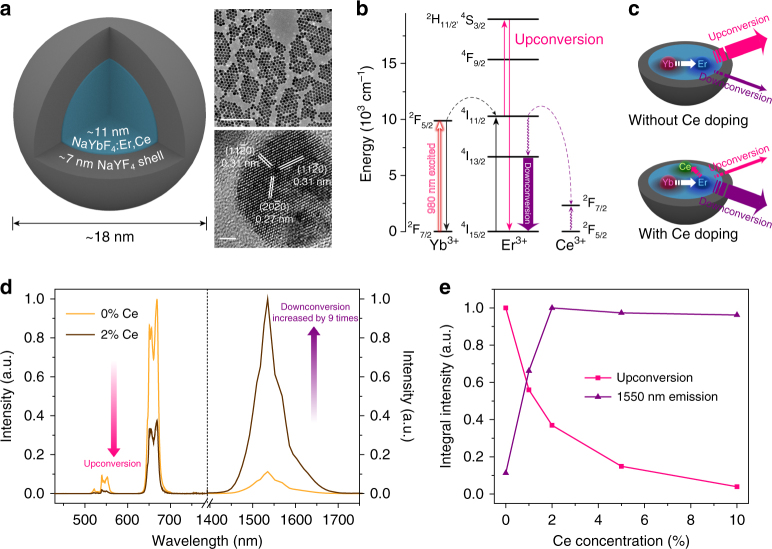
Ce^3+^ doped rare-earth nanoparticles with enhanced NIR-IIb luminescence. **a** Schematic design of a NaYbF_4_:Er,Ce@NaYF_4_ core-shell nanoparticle (left) with corresponding large scale TEM image (upper right, scale bar = 200 nm) and HRTEM image (lower right, scale bar = 2 nm). **b** Simplified energy-level diagrams depicting the energy transfer between Yb^3+^, Er^3+^, and Ce^3+^ ions. **c** Schematic illustration of the proposed energy-transfer mechanisms in Er-RENPs with and without Ce^3+^ doping. **d** Upconversion and downconversion luminescence spectra of the Er-RENPs with 0 and 2% Ce^3+^ doping. **e** Schematic representation of Ce^3+^ doping concentration and corresponding upconversion and downconversion emission intensity of the Er-RENPs upon 980 nm excitation

A thermal decomposition method^26^ was used to synthesize the NaYbF_4_:2%Er,2%Ce@NaYF_4_ NPs by co-thermolysis of rare-earth trifluoroacetates in oleic acid, 1-octadecene, and/or oleylamine to produce uniform spherical morphology and narrow size distribution (Supplementary Methods). Er-RENPs with an overall size of ~ 18 nm were synthesized, comprised of a Ce^3+^ and Er^3+^ co-doped NaYbF_4_ core surrounded by a NaYF_4_ passive shell of ~ 7 nm (Fig. 1a). To generate 1550 nm luminescence from Er-RENPs, Yb^3+^ ions were used as sensitizers to harvest 980 nm photons by pumping electrons to populate the ^2^F_5/2_ state of Yb^3+^. Efficient Yb^3+^ → Er^3+^ energy transfer ensured Er^3+^ excitation to the intermediate ^4^I_11/2_ level (Fig. 1b). In the UC emission process, Er^3+^ ions possessing adequate long-lived intermediate ^4^I_11/2_ state were excited further to higher ^2^H_11/2_ and ^4^S_3/2_ energy levels followed by the UC luminescence (Fig. 1b). For NIR-IIb DC emission, a short lived ^4^I_11/2_ excited state lifetime would allow a rapid nonradiative decay into the ^4^I_13/2_ level followed by 1550 nm DC emission. This could also transform the otherwise reversible Yb^3+^ → Er^3+^ energy transfer process into an irreversible one^27^.

To shorten the Er ^4^I_11/2_ lifetime, enhance the down-conversion pathway and suppress upconversion, we doped into the nanoparticle core Ce^3+^ ions whose energy-level diagram presenting only two levels (Fig. 1c). The energy spacing between ^2^F_5/2_ and ^2^F_7/2_ levels of Ce^3+^ is about 2300 cm^−1^ (ca. 0.29 eV), which provides a small mismatch with the ^4^I_11/2_−^4^I_13/2_ energy difference (~ 3700 cm^−1^; ca. 0.46 eV) of Er^3+^, allowing efficient non-radiative phonon-assisted cross relaxation of the Er ^4^I_11/2_ state^28^. Note that this concept was used in Ce/Er/Yb co-doped phosphate glass to enhance 1550 nm luminescence for laser applications previously^29, 30^. The Er ^4^I_13/2_ level is significantly populated through the accelerated nonradiative relaxation of Er ^4^I_11/2_ → ^4^I_13/2_ facilitated by the Ce^3+^ dopants, resulting in an impressive 9-fold enhancement of the down-conversion 1550 nm luminescence of the Er-RENPs (Fig. 1d). In contrast, the UC emission of the Er-RENPs at 540 and 650 nm are both dramatically decreased, due to the depopulation of the UC emissive ^2^H_11/2_, ^4^S_3/2_, and ^4^F_9/2_ levels in the presence of Ce^3+^ ions (Supplementary Fig. 1).

Ideally the population of ^4^I_13/2_ emitting level should be further improved by increasing the Ce^3+^ concentration. However, we found that the 1550 nm luminescence ceased to increase under higher Ce^3+^ doping concentrations (Fig. 1e), indicating a limit to the ^4^I_11/2_ state desensitization by Ce^3+^ions. The resulting 2% Ce doped Er-RENPs (emission 1500–1700 nm; excitation 980 nm, 10 W cm^−2^) was much brighter than the previous record^24^ evident from much shorter exposure times (20 ms) for in vivo imaging. Note that we found a nonlinear relationship between the down-conversion emission and excitation power *p*, following a *p* ^0.734^ power law relationship while UC following a *p* ^1.561^ power law relationship (Supplementary Fig. 3). Such non-linearity should always be considered for rare-earth based luminescence involving multi-photons.

### Surface modification of Er-RENPs for biocompatibility

**Fig. 2:**
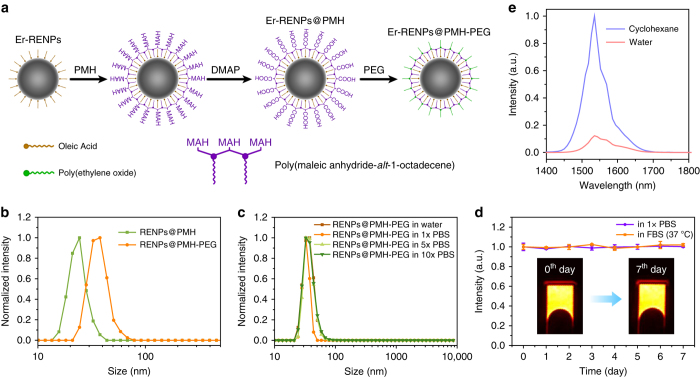
Surface modification of the Er-RENPs. **a** Schematic illustration outlining the PMH coating and PEGylation procedure for the Er-RENPs (Er-RENPs@PMH-PEG). **b** DLS spectra of PMH capped RENPs before and after PEGylation. **c** DLS spectra demonstrating the well dispersibility of RENPs@PMH-PEG in different concentration of PBS solution. **d** Downconversion emission intensity of Er-RENPs@PMH-PEG in 1x PBS and 37 °C FBS solution as a function of days. The inset showed 1550 nm luminescence images of Er-RENPs@PMH-PEG in 1x PBS at 0th and 7th day. **e** Downconversion luminescence spectra of oleic acid-capped Er-RENPs dispersed in cyclohexane and Er-RENPs@PMH-PEG dispersed in water

For in vivo biological imaging, a hydrophilic surface must be imparted to the Er-RENPs for high dispersibility and stability in aqueous solutions. Various strategies have been established to convert rare-earth nanocrystals from hydrophobic to hydrophilic including ligand oxidation^31^, ligand free^32^, ligand exchange^33^, and ligand interaction^34^ methods. Here, we created a hydrophilic polymer shell on the surface of Er-RENPs by exploiting simple van-der-waals interactions between the alkyl chains of poly(maleic anhydride-*alt*-1-octadecene) (PMH; average molecular weight: 30,000–50,000) and the oleic acid molecules on the RENPs (Fig. 2a).

In the first step, PMH and the oleic acid coated nanoparticles were mixed and stirred in chloroform to allow insertion for alkyl chains on PMH into the oleic acid coating on Er-RENPs. After evaporating the solvent, an aqueous solution of 4-(dimethylamino)pyridine (DMAP) was added to re-disperse the nanoparticles through sonication. The DMAP served as nucleophilic catalyst for the esterification with the outward anhydride groups of PMH^35, 36^. Each of the anhydride rings on PMH was transformed into two carboxyl groups, thus rendering the Er-RENPs water dispersible (Fig. 2a). Dynamic light scattering (DLS) measurements showed an average hydrodynamic radius (*R*_H_) of ~ 26 nm for the PMH capped Er-RENPs in pure water (Fig. 2b), corresponding to the 18 nm sized RENPs with oleic acid and PMH coating layers. To render the RENPs more biocompatible, we performed a further PEGylation step through conjugation of methoxy polyethylene glycol amine (mPEG-NH_2_; average molecular weight: 5000) onto the nanoparticles. DLS measurements (Fig. 2b) showed that the average *R*_H_ of the RENPs increased by 11 nm after PEGylation. The PEGylated Er-RENPs@PMH^−^PEG showed excellent dispersibility in high-salt solutions (Fig. 2c), with no detectable aggregation even in 10x PBS buffer solution. High aqueous dispersion stability (Fig. 2d; Supplementary Fig. 5) and photostability of the PEG-RENPs in PBS and fetal bovine serum (Supplementary Fig. 6) were also confirmed. However, the luminescence intensity of Er-RENPs in aqueous solution seriously decreased compared with that in cyclohexane (Fig. 2e).

### Reducing the quenching effect to the Er-RENPs in aqueous solution

**Fig. 3:**
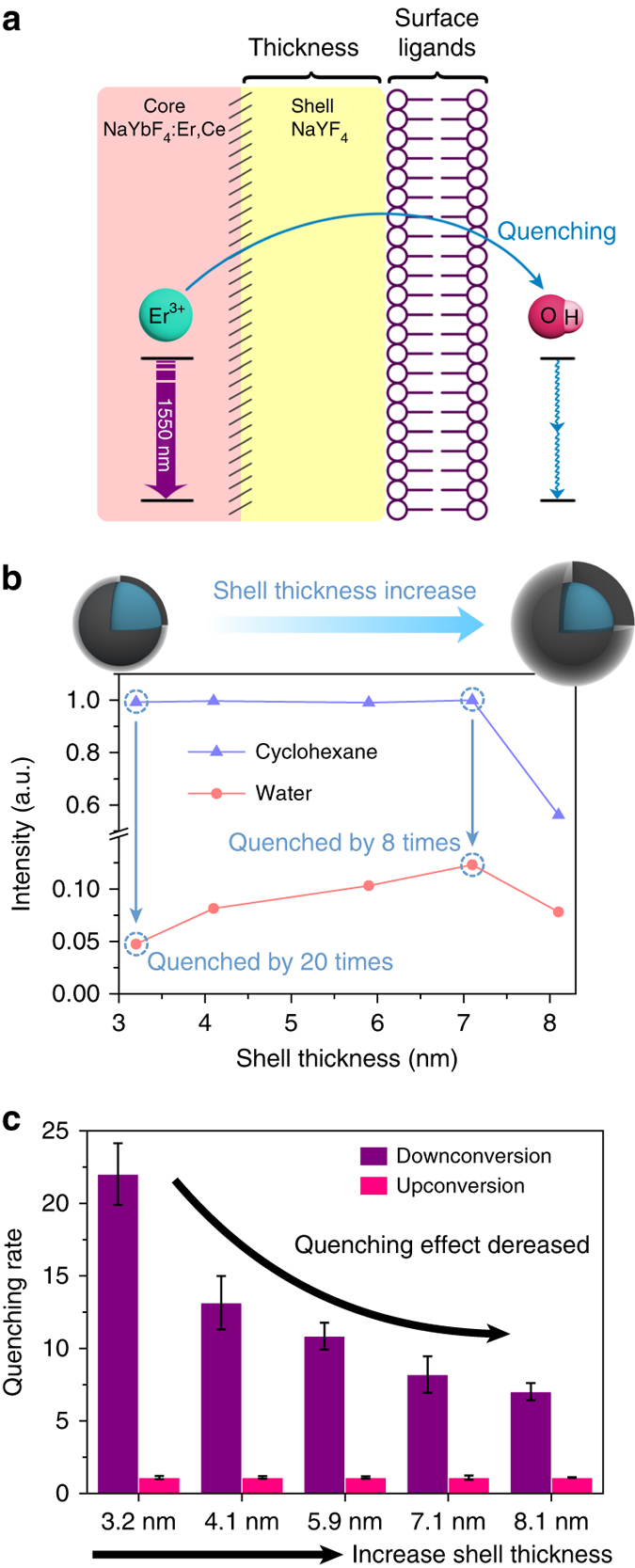
Reducing aqueous quenching effect by controlling the inert shell thickness of Er-RENPs. **a** Schematic illustration of the proposed quenching mechanisms of Er-RENPs in aqueous solution. **b** Schematic representation of shell thickness and corresponding 1550 nm downconversion emission intensity of the Er-RENPs in organic and aqueous phase upon 980 nm excitation. **c** Quenching rate of upconversion and downconversion emission as a function of shell thickness (from 3.2 nm to 8.1 nm). Three surface coating experiments were performed for each Er-RENPs sample. All data are presented as means ± s.d

Hydroxyl group has been shown to be a serious quencher for the DC emission of Er^3+^ ^37–39^. The energy spacing of Er^3+ 4^I_13/2_ → ^4^I_15/2_ transition for the 1550 nm luminescence is ~ 6500 cm^−1^, giving way to a two-phonon quenching mechanism by the OH^−^ groups (Fig. 3a) with fundamental stretching vibration frequencies in the range of 2700–3700 cm^−1^. In fact, this drastic quenching effect is reported to be more than 20 times stronger than that of the Yb^3+^ ^22^, causing a remarkable intensity decrease of the 1550 nm emission after hydrophilic coating of Er-RENPs for aqueous solubility and compatibility (Fig. 3b)^40^.

To reduce the aqueous quenching effect, the most common way is coating a NaYF_4_ passive shell to increase the distance between lanthanide ions and surface quenchers^41^. Usually an additional NaYF_4_ layer of <3 nm thick is adequate to protect the UC luminescence from quenching effect originating from surface ligands and aqueous solvents^42,43^. Indeed, when we synthesized a ~ 3 nm NaYF_4_ shell on the Ce doped NaYbF_4_:2%Er,2%Ce nanoparticles (confirmed by transmission electron microscopy (TEM), Supplementary Fig. 7a, b), we retained more than 90% of the UC emission intensity of the Er-RENPs after aqueous transfer (Supplementary Fig. 9). However, we found that the 1550 nm DC emission intensity of these Er-RENPs decreased by as much as 20-fold (defined as the quenching rate) after transferring to water (Fig. 3b, c). This led us to grow thicker inert shells up to 8 nm to further isolate the Er^3+^ ions in the core of the nanoparticle from water (see Supplementary Fig. 7 for TEM; see Supplementary Fig. 10 for DLS analysis). A gradual increase in the NIR-IIb emission of the Er-RENPs in water solution was observed as the shell thickness increased (Fig. 3b). When the NaYF_4_ shell thickness was controlled at ~ 7 nm, the 1550 nm emission intensity of the Er-RENPs reached a maximum, which was 2.5 times brighter than the Er-RENPs with 3 nm shell. Further increase in the thickness of NaYF_4_ shell afforded no further enhancement of the 1550 nm luminescence (Fig. 3b), likely due to reduced absorption of the excitation light by Yb^3+^ in the core through a thicker shell^44^. Indeed, thicker shells lowered the DC and UC luminescence alike in both organic and aqueous solution (Supplementary Fig. 9). This was the first investigation of the effects of inert shell thickness on the DC NIR-IIb luminescence of the core-shell Er-RENPs.

In terms of absolute QY, due to discrepancies in the reported QY of the IR-26 reference fluorophore (QY_IR26_: ~ 0.05–0.5%)^45, 46^, the absolute QYs of our Ce doped Er-RENP in aqueous solutions were estimated to be in the range of 0.27–2.73% (Supplementary Fig. 12) under the laser excitation of 10 W cm^−2^. Although this was the highest among reported down-conversion RENPs, the 1550 nm luminescence of our Er-RENPs was still seriously quenched by 8 times in the water phase relative to in cyclohexane (Fig. 3b, c), and remains a challenge to be further addressed.

### Fast in vivo cerebrovascular imaging in NIR-IIb window with RENPs

**Fig. 4:**
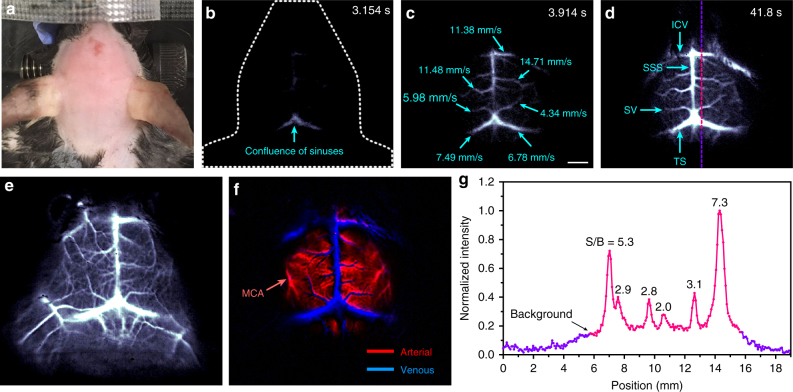
Fast in vivo brain imaging with Er-RENPs@PMH-PEG in the NIR-IIb region. **a** Color photograph of a C57Bl/6 mouse (with hair shaved off) preceding NIR-IIb fluorescence imaging. **b**–**d** Time-course NIR-IIb brain fluorescence images (exposure time: 20 ms) showing the perfusion of RENPs into various cerebral vessels. The blood-flow velocities of cerebral vessels are given in **c** (scale bar corresponds to **b**–**d**: 2 mm). **e**, **f** Cerebral vascular image (exposure time: 20 ms) in NIR-IIb region with corresponding PCA overlaid image **f** showing arterial (red) and venous (blue) vessels. **g** SBR analysis of NIR-IIb cerebrovascular image **d** by plotting the cross-sectional intensity profiles

With the bright Ce doped Er-RENPs and a 2D InGaAs camera (Princeton Instruments, detection range 800–1700 nm), we performed in vivo mouse brain vessel imaging (Fig. 4) by exciting the Er-RENPs with a 980 nm laser while detecting the 1550 nm luminescence of intravenously injected Er-RENPs@PMH-PEG solution. Imaging was done through the intact mouse scalp and skull in a non-invasive manner. Benefiting from the bright luminescence of Ce doped Er-RENPs, we were able to carry out dynamical imaging and tracking of arterial blood flow in the mouse brain in the 1500–1700 nm NIR-IIb window using a much shorter exposure time (20 ms) than previously possible (200–5000 ms) using rare-earth materials^25^, carbon nanotubes^15^, and QD^10^ NIR-IIb probes.

Immediately after tail vein injection of 200 μl solution of Er-RENPs@PMH-PEG at a concentration of ~ 28 mg/ml, video-rate imaging of the mouse brain (C57Bl/6 mouse) was performed (using a 980 nm laser excitation and luminescence detection in the 1500–1700 nm range) with each frame recorded under a 20 ms exposure time. With the excellent temporal resolution, NIR-IIb emission in the confluence of sinuses was observed within ~ 3 s post-injection (Fig. 4a, b; Supplementary Movie 1). At ~ 4 s post-injection, blood flow into the inferior cerebral veins (ICV), the superior sagittal sinus (SSS), the superficial veins (SV), and the transverse sinus (TS) began to show up (Fig. 4c). By plotting the distance traveled by the flow front as a function of time, we were able to obtain a spatially resolved blood-flow map in the brain (Fig. 4c; Supplementary Fig. 13; blood-flow velocity in a range of ~4.34–14.71 mm s^−1^). This was the first time that video-rate NIR-IIb through-skull imaging of mouse cerebral vessels is sufficiently fast to image and quantify cerebral blood-flow velocities.

Principal component analysis (PCA) of the dynamic images was performed by time-coursing 80 frames over a time course of ~3 s (Fig. 4e, f)^47^. Various venous vessels (blue, Fig. 4f) including the ICVs, the SSS, the SV, and the TS were discriminated from the arterial vessels (red, Fig. 4f) of the middle cerebral artery (MCA).

Within tens of seconds post injection, the Er-RENPs@PMH-PEG fully perfused into the mouse brain vessels and clearly outlined cerebral vascular structures at a depth of >1.3 mm under the intact scalp and skull (Fig. 4d). By plotting the cross-sectional intensity profiles, we measured the vessel signal-to-background ratios to be 5.3 for the ICVs and 3.1 for the SV (Fig. 4g), compared with previous results of 4.5 for the ICVs^15^ and 1.49 for the SV obtained with dimmer QDs^10^. Note that the total thickness of scalp skin and skull is ~ 1.3 mm^6^ for the mouse. The high degree of image clarity and high signal/background of cerebral-vasculatures recorded with RENPs in the NIR-IIb window (see Supplementary Figs. 15 and 16 for detailed phantom studies) using 20 ms exposure time confirmed the development of an advanced, bright, biocompatible luminescent probe based on rare-earth materials.

Over a period of 48 h post injection, Er-RENPs were observed to accumulate in the liver and spleen (Supplementary Fig. 17a), suggesting uptake by cells of the reticuloendothelial system^48, 49^. The strong NIR-IIb emission in the feces collected (Supplementary Fig. 17c) indicated excretion of the Er-RENPs by the hepatobiliary route, resulting in a dramatical intensity decrease of the liver signal at 44 days post injection to a low accumulation of only ~ 6%, suggesting high degree of excretion of the nanoparticles (Supplementary Fig. 17). No obvious toxicity was observed with mice injected with the RENPs, indicating the potential clinical imaging application of Er-RENPs due to their high biocompatibility and chemically inert, though a systematic investigation of this topic will be needed before any clinical application in human trials.

## Discussion

Down-conversion NIR-IIb emissive rare-earth Er-based nanoparticles were engineered to enhance the 1550 nm luminescence through Ce^3+^ doping and optimization of the inert shell coating. These led to a bright Er-RENP with a 1550 nm emission under 980 nm excitation in aqueous solution. Owing to the strong 1550 nm emission of the Er-RENPs, fast imaging of mouse cerebral-vasculatures in NIR-IIb window was achieved with a short exposure time of 20 ms per frame and high spatiotemporal resolution. Such non-invasive NIR-IIb imaging could facilitate real-time monitoring and visualization of cerebrovascular abnormalities toward the diagnosis and therapy of the cerebral diseases.

## Methods

### Synthesis of *β*-NaYbF_4_:Ce,Er@NaYF_4_ nanoparticles

In a typical four-step synthetic procedure, 1 mmol of CF_3_COONa and 1 mmol of RE(CF_3_COO)_3_ [RE: 96% Yb, 2% Ce, 2% Er] were added to a mixture of OA (10 mmol; oleic acid), OM (10 mmol; oleylamine), and ODE (20 mmol; 1-octadecene) in a two-necked flask at room temperature. The solution was pre-degassed for 30 min with vigorous magnetic stirring then heated to 120 °C under vacuum for 30 min to remove water and oxygen. The solution was then heated to 325 °C at 10 °C/min and maintained for 1 h under argon protection. After cooling to room temperature, an excess amount of ethanol was poured into the solution. The resultant nanocrystals were centrifuged at 4400 rpm for 30 min, washed with ethanol several times, and dispersed in 2 ml of cyclohexane. The second step was similar to above procedure, except that 1 mmol CF_3_COONa, 1 mmol of Yb(CF_3_COO)_3_ and the prepared nanocrystals were added to a mixture of OA (20 mmol) and ODE (20 mmol) and maintained at 305 °C for 75 min and 310 °C for another 20 min under argon protection. Repeat the second step two more times; and the final resultant nanoparticles (oleic acid-capped) were dispersed in 3 ml of cyclohexane for further hydrophilic treatment.

### Preparation of PMH coated rare-earth nanoparticles

The as-prepared oleic acid-capped RENPs (20 mg) were dried at 60 °C to evaporate cyclohexane and then dissolved in 1 ml chloroform. 80 mg PMH (30–50 kDa; poly(maleic anhydride-*alt*-1-octadecene); Sigma-Aldrich) dissolved in 3 ml chloroform was then added; and the mixed solution was stirred for overnight. Chloroform was then evaporated by rotovap. 40 mg DMAP dissolved in 5 ml water was added to re-disperse the residue. The residue was sonicated for 30 min at room temperature to form a clear RENPs@PMH solution.

### Preparation of PEGylated RENPs@PMH nanoparticles

The above RENPs@PMH solution were centrifuged (15,000 rpm, 1.5 h) and washed with water two times to remove excess PMH and DMAP. The afforded RENPs@PMH were then dispersed in 2 ml MES solution (10 mM; 4-Morpholineethanesulfonic acid; the pH was tuned to 6.5 with sodium hydroxide solution). 4 mg mPEG-NH_2_ (5 kDa; Methoxy polyethylene glycol amine; Laysan Bio) dissolved in 2 ml MES solution (10 mM; pH = 6.5) was added into above solution and shaken for 10 min. 2 mg 1-e﻿thyl-3-(3-dimethylaminopropyl)carbodiimide hydrochlorid﻿e (EDC) dissolved in 200 μl water was then added; and the solution was shaken for 3 h. 20 μl Tris-HCl solution (1 M; Thermo Fisher Scientific) was added; and the solution was shaken for another 1 h. The solution was centrifuged at 4400 rpm for 30 min; and the supernate containing RENPs@PMH-PEG was washed with centrifugal filter (100 K) for 2 times to remove excess EDC and mPEG-NH_2_. The afforded RENPs@PMH-PEG were ready to disperse in water, PBS solution, and FBS solution.

### Mouse handling

All vertebrate animal experiments were performed under the approval of the Stanford University’s Administrative Panel on Laboratory Animal Care. C57Bl/6 male mice were obtained from Taconic Farms. Before brain and hindlimb vessel imaging, a rodent anesthesia machine with 2 l min^−1^ O_2_ gas flow mixed with 2.5% Isoflurane was used to anaesthetize the mice. The hair over the scalp and hindlimb skin was carefully removed using Nair to avoid causing wounds to the skin. Tail vein injection of the RENPs@PMH-PEG contrast agent was carried out in dark and synchronized with the camera that started continuous image acquisition simultaneously. For brain and hindlimb imaging in the NIR-IIb window, a 1x PBS solution (200 μl) of 28 mg/ml RENPs@PMH-PEG was injected. During the dynamic imaging the mouse was kept anaesthetized by a nose cone delivering 2 l·min^−1^ O_2_ gas mixed with 2.5% Isoflurane. The sample sizes of mice were selected based on previously reported studies. No blinding was performed. Mice were randomly selected from cages for all experiments. All groups within study contained *n* = 5 mice.

### Dynamic fluorescence imaging in the NIR-IIb window

A liquid-nitrogen-cooled, 320 × 256 pixel two-dimensional InGaAs array (Princeton Instruments) was used to carry out in vivo imaging of mouse brain and hindlimb. The excitation light was provided by a 980 nm continuous-wave laser coupled to a collimator (F240SMA-980; Thorlabs). The excitation power density at the imaging plane was 150 mW cm^−2^. The emitted fluorescence was allowed to pass through a 1100 nm and a 1500 nm long-pass filter (Thorlabs) to ensure the NIR images taken in the NIR-IIb region of 1500–1700 nm. The upper bound at 1700 nm was determined by the sensitivity profile of the InGaAs detector. A lens pair consisting of two achromats (200 and 75 mm; Thorlabs) was used to focus the image onto the detector with a field of view of 25 × 20 mm. The exposure time for each image acquisition was 20 ms, while the overhead time of the camera is ~ 19 ms. Therefore, the frame rate we used for dynamic imaging is 1/(20 + 19 ms) = 25.6 Hz (the temporal resolution is 39 ms). To perform PCA, early image frames immediately after injection (200 μl of RENPs@PMH-PEG at a concentration of 28 mg/ml) were loaded into an array using MATLAB software^6^.

### Data availability

The data that support the findings of this study are available from the corresponding authors upon reasonable request.

## Electronic supplementary material

Supplementary Information

Description of Additional Supplementary Information

Supplementary Movie 1

Supplementary Movie 2
