# Supplementary material for: Boosting the down-shifting luminescence of rare-earth nanocrystals for biological imaging beyond 1500 nm
Source: Nat Commun. 2017 Sep 29;8:737. doi: 10.1038/s41467-017-00917-6 (PMC5622117; doi:10.1038/s41467-017-00917-6)
Supplement: Supplementary file 1 — Supplementary Information [file 41467_2017_917_MOESM1_ESM.pdf]

Supplementary Methods

Reagents. Rare-earth(III) oxides (RE: Y, Yb, Er), cerium(III) nitrate, trifluoroacetic acid (99%) and chloroform were purchased from Alfa Asea and used as received. CF_3COONa, cyclohexane, 1-(3-dimethylaminopropyl)-3-ethylcarbodiimide hydrochloride (EDC), Oleic acid (OA), oleylamine (OM), 1-octadecene (ODE), 4-Morpholineethanesulfonic acid (MES), poly(maleic anhydride-*alt*-1-octadecene) (PMH; 30-50 kDa), 4-(dimethylamino)pyridine (DMAP) and sodium carbonate were purchased from Sigma-Aldrich and used without further purification. Methoxy polyethylene glycol amine (mPEG-NH_2; 5000 m.W.) were purchased from Laysan-Bio. Phosphate Buffered Saline (10x PBS) was purchased from HyClone. $RE(CF_3COO)_3$ were prepared by the literature method.[1]

Preparation of $Ce(CF_3COO)_3$. 0.636 g of Na_2CO_3 dissolved in 10 ml water was added into 40 ml $Ce(NO_3)_3$ aqueous solution containing 1.736 g of $Ce(NO_3)_3$. Large amount of precipitate indicated the generation of $Ce_2(CO_3)_3$. The solution was centrifuged and washed with water 4 times to remove excess Na_2CO_3 and $Ce(NO_3)_3$. After that, 4 ml trifluoroacetic acid was added and the solution was stirred for 3 hours at 80 ^oC. Then 4 ml water was added and the solution was stirred for another 3 hours at 80 ^oC. The solution was then evaporated by rotavap to obtain $Ce(CF_3COO)_3$ powder with further drying at 60 ^oC for 6 hours.

Synthesis of β-$NaYbF_4$:Ce,Er (core, ~ 11 nm) nanoparticles. In a typical two-step synthetic procedure of β-$NaYbF_4$:Ce,Er, 1 mmol of CF_3COONa and 1mmol of $RE(CF_3COO)_3$ [RE: 96% Yb, 2% Ce, 2% Er] were added to a mixture of OA (10 mmol), OM (10 mmol), and ODE (20 mmol) in a two-necked flask at room temperature. The solution was pre-degassed for 30 minutes with vigorous magnetic stirring then heated to 120 ^oC under vacuum for 30 minutes to remove water and oxygen, and thus to form an optically transparent solution. The solution was then heated to 325 ^oC at 10 ^oC/min and maintained for 1 hour under argon protection. After cooling to room temperature, an excess amount of ethanol was

poured into the solution. The resultant nanocrystals was centrifuged at 4400 rpm for 30 minutes, washed with ethanol several times, and dispersed in 2 ml of cyclohexane. The second step was similar to above procedure, except that 1 mmol CF_3COONa, 1mmol of $Yb(CF_3COO)_3$ and the prepared nanocrystals were added to a mixture of OA (20 mmol) and ODE (20 mmol) in a two-necked flask and maintained at 305 ^oC for 75 minutes and 310 ^oC for another 20 minutes under argon protection. The afforded nanoparticles (~ 11 nm) were dispersed in 2 ml of cyclohexane for further coating.

Synthesis of $NaYbF_4$:Ce,Er@$NaYF_4$ (core-shell, ~ 18 nm) nanoparticles. In a typical two-step synthetic procedure of $NaYbF_4$:Ce,Er@$NaYF_4$ with ~ 7 nm shell, an amount of 1 mmol CF_3COONa, 1 mmol $Y(CF_3COO)_3$ and the prepared β-$NaYbF_4$:Ce,Er (core) nanocrystals were added to a mixture of OA (20 mmol), ODE (20 mmol) in a two-necked flask at room temperature. The solution was pre-degassed for 30 minutes with vigorous magnetic stirring then heated to 120 ^oC under vacuum for 30 minutes to remove water and oxygen. The solution was then heated to 305 ^oC for 75 minutes and 310 ^oC for another 20 minutes under argon protection. After cooling to room temperature, an excess amount of ethanol was poured into the solution. The resultant nanocrystals (~ 3 nm shell) was centrifuged at 4400 rpm for 30 minutes, washed with ethanol several times, and dispersed in 2 ml of cyclohexane. The synthetic procedure of $NaYbF_4$:Ce,Er@$NaYF_4$ with ~ 7 nm shell was similar to above procedure, except that 1 mmol CF_3COONa, 1 mmol $Y(CF_3COO)_3$ and 1/3 of above prepared $NaYbF_4$:Ce,Er@$NaYF_4$ nanocrystals were added to a mixture of OA (20 mmol) and ODE (20 mmol) in a two-necked flask and maintained at 305 ^oC for 75 minutes and 310 ^oC for another 20 minutes under argon protection. The resultant nanocrystals (~ 7 nm shell) was centrifuged at 4400 rpm for 30 minutes, washed with ethanol several times, and dispersed in 3 ml of cyclohexane. The concentration of the RENPs was measured by fully drying the nanoparticles in a 60 ^oC oven followed by careful weighing.

Preparation of PMH coated rare-earth nanoparticles (RENPs@PMH). The as-prepared oleic acid-capped RENPs (20 mg) were dried at 60 ^{o}C to evaporate cyclohexane and then dissolved in 1 ml chloroform. 80 mg PMH was dissolved in 3 ml chloroform. The above solutions were mixed and stirred for overnight. Chloroform was then evaporated by rotovap. 5 ml aqueous solution containing 40 mg DMAP was added to re-disperse the residue. The residue was sonicated for 30 minutes at room temperature to form a clear solution.

PEGylation of PMH coated rare-earth nanoparticles (RENPs@PMH-PEG). The RENPs were washed by centrifugation for three times (15000 rpm; 1.5 hours) to remove excess PMH and DMAP. Above RENPs@PMH were then dispersed in 2 ml MES solution (10 mM; pH=6.5). 4 mg mPEG-NH_2 dissolved in 2 ml MES solution (10 mM; pH=6.5) was added into above solution and shaken for 10 minutes. 2 mg EDC dissolved in 200 μl water was then added; and the solution was shaken for 3 hours. 20 μl Tris-HCl solution (1 M; Thermo Fisher Scientific) was added; and the solution was shaken for another 1 hour. The solution was centrifuged at 4400 rpm for 30 minutes; and the supernate was washed with centrifugal filter (100K) for 2 times. The afforded RENPs@PMH-PEG were ready to disperse in water, PBS solution and FBS solution.

Dynamic fluorescence imaging in the NIR-IIb window. A liquid-nitrogen-cooled, 320 × 256 pixel two-dimensional InGaAs array (Princeton Instruments) was used to carry out *in vivo* imaging of mouse brain and hindlimb. The excitation light was provided by a 980 nm continuous-wave (CW) laser coupled to a collimator (F240SMA-980; Thorlabs). The diameter of the 980 nm laser beam was 2.8 cm at the imaging plane; the excitation power density was 150 mW·cm^{-2}. According to the International Commission on Non-ionizing Radiation Protection[2], the safe laser exposure limit for the skin at 980 nm: $EL_{980} = 0.2 \times C_A$ W·cm^{-2} = 726 mW·cm^{-2} ($C_A = 10^{[0.002\,(\lambda - 700)]}$ if λ = 700 - 1050 nm and the exposure duration t = 10 - 30000 s). The laser exposure duration in our experiments was below 60 s; and the

excitation power density was 150 mW·cm^{-2} which is much lower than the safe exposure limit of 726 mW·cm^{-2} at 980 nm. The emitted fluorescence was allowed to pass through a 1100 nm and a 1500 nm long-pass filter (Thorlabs) to ensure the NIR images taken in the NIR-IIb region of 1500-1700 nm. The upper bound at 1700 nm was determined by the sensitivity profile of the InGaAs detector. A lens pair consisting of two achromats (200 mm and 75 mm; Thorlabs) was used to focus the image onto the detector with a field of view of 25 mm × 20 mm. The exposure time for each image acquisition was 20 ms, while the overhead time of the camera is ~ 19 ms. Therefore, the frame rate we used for dynamic imaging is 1 / (20 ms + 19 ms) = 25.6 Hz (or 25.6 frames per second). To perform principal-component-analysis (PCA), early image frames immediately after injection (200 μl of RENPs@PMH-PEG at a concentration of 28 mg/ml) were loaded into an array using MATLAB software.[3]

Characterization. Transmission electron microscopy (TEM) images and high-resolution TEM images were taken with a JEM-2100F transmission electron microscope (JEOL) operating at 200 kV. Dynamic light scattering (DLS) measurements were performed on a Malvern Zetasizer Nano ZS90. X-ray diffraction (XRD) patterns were recorded on a Philips XPert PRO MPD X-ray diffractometer operated at 35 kV and 45 mA with Cu-Kα radiation. The upconversion luminescent properties were studied using a Horiba Jobin Yvon FluoroLog3 spectrometer equipped with a 980 nm CW laser as excitation. The downconversion luminescent properties were studied using a Acton SP2300i spectrometer equipped with an InGaAs linear array detector (Princetion OMA-V) and using a 980 nm CW laser as excitation. NIR fluorescence images of the downconversion emission were obtained using 2D InGaAs array (Princeton Instruments) with 320 × 256 pixel using 980 nm CW laser as excitation.

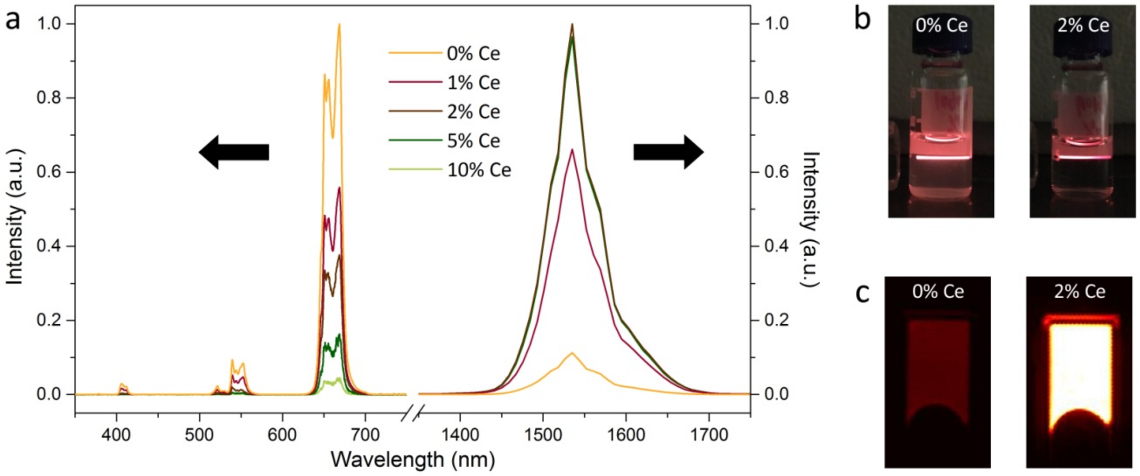

Supplementary Figure 1: Influence of Ce doping on the luminescence of Er-RENPs. (**a**) Upconversion and downconversion luminescence spectra of Er-RENPs with different Ce doping concentration. (**b**) Upconversion luminescence photographs of Er-RENPs with and without Ce doping. (**c**) Downconversion luminescence images of Er-RENPs with and without Ce doping. The nanoparticles were excited by a 980 nm CW laser.

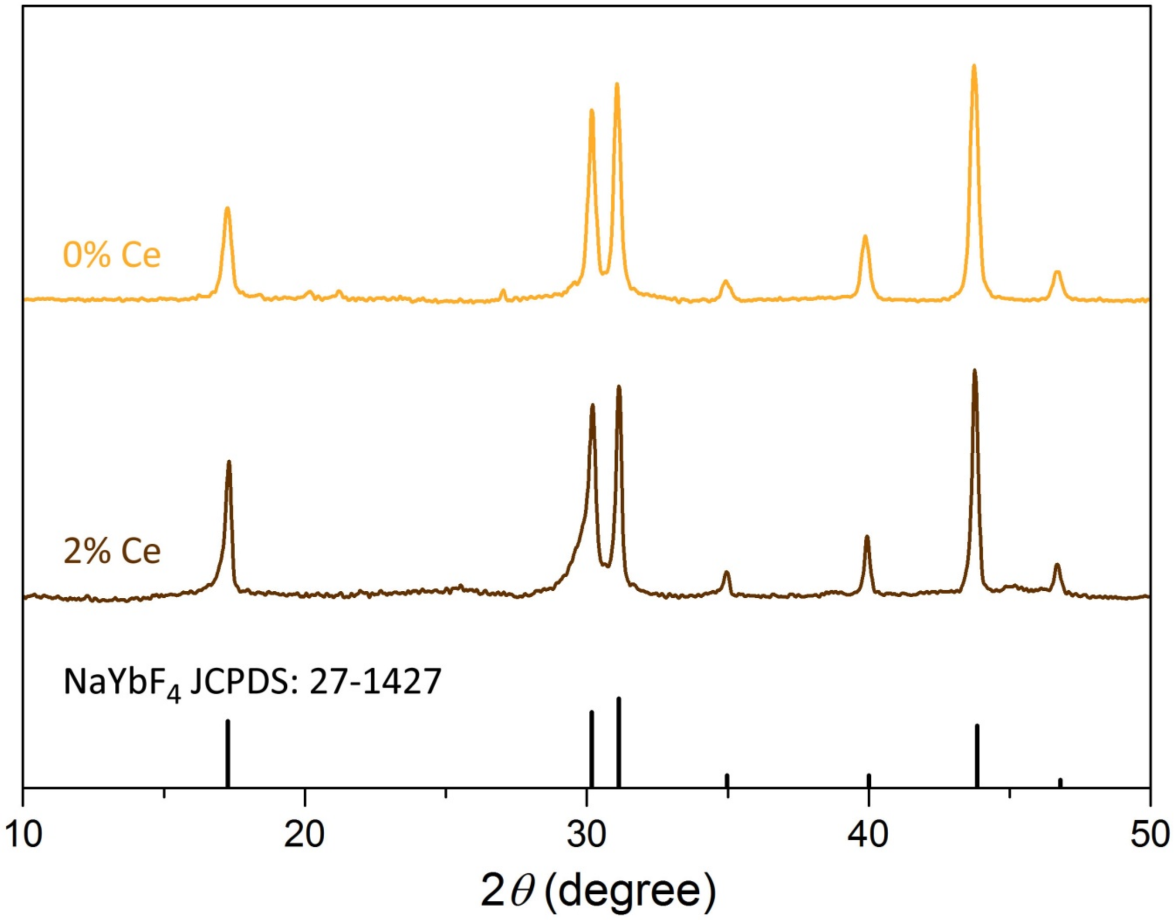

Supplementary Figure 2: XRD measurements. XRD patterns of Er-RENPs with and without Ce doping (JCPDS no. 27-1427 for hexagonal β-$NaYbF_4$ crystalline structure). Note that the luminescence of rare-earth doped nanoparticles is highly influenced by the crystalline structure. Our results showed similar XRD patterns of Er-RENPs with and without Ce doping, indicating that the enhanced downconversion luminescence was not caused by the crystalline structure changes after Ce doping.

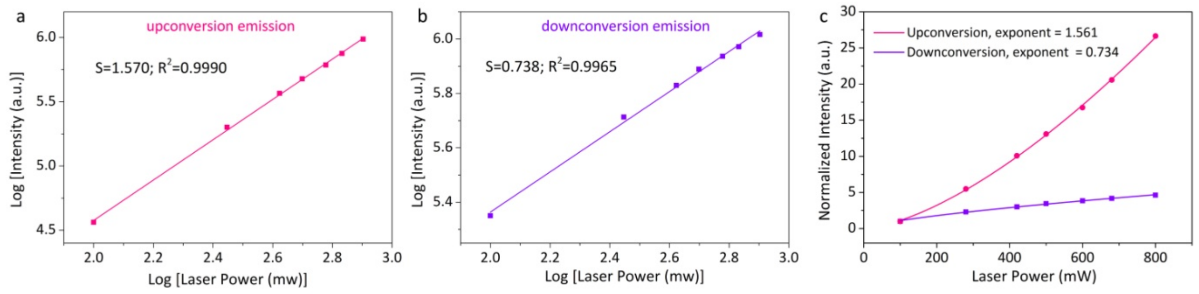

Supplementary Figure 3: Excitation power dependent upconversion and downconversion emission of Er-RENPs. To determine the number of photons involving in the upconversion and downconversion luminescence, the emission intensities of the Er-RENPs were recorded as a function of the 980 nm laser density in log-log plots.[4] From **a**, the slope for the upconversion luminescence was 1.570 (in the range of 1-2), indicating a two-photon emission process. From **b**, the slope for the downconversion luminescence was 0.738 (in the range of 0-1), indicating a one-photon emission process. Furthermore, the nonlinear relationship between the up-, down-conversion emission and laser power was expressed as a power law equation: $I = k \times P^a$, where I is the emission intensity, P is the laser power, a is the scaling exponent (**c**). The obtained exponents of the upconversion (1.561) and downconversion (0.734) emission were consistent with the slopes from log-log plots (**a, b**).

It is important to note that the quantum yield of rare-earth nanoparticles is dependent on the excitation power density due to this nonlinearity of the upconversion and downconversion processes.[5] Such non-linearity should always be considered for rare-earth based luminescence involving multi-photons.

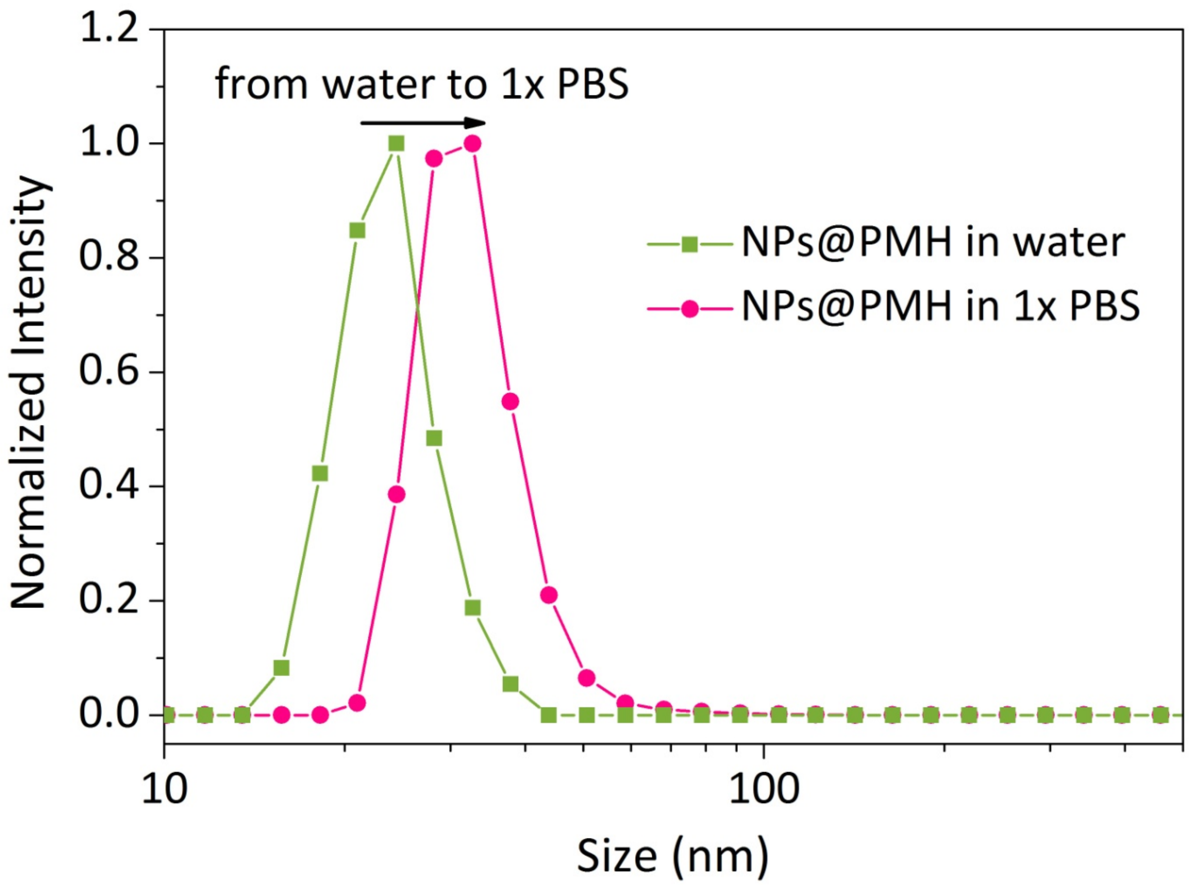

Supplementary Figure 4: DLS measurements of the Er-RENPs@PMH in water and 1x PBS solution. Although the PMH coated Er-RENPs were stable in 1x PBS solution, the DLS measurements showed that the hydrodynamic radius of the nanoparticles increased from ~ 26 nm to ~ 33 nm after transferral from water to 1x PBS solution, indicating the aggregation of the Er-RENPs@PMH in saline solution. According to our experiments, the intravenously injection of these Er-RENPs@PMH into the mouse can cause blood vessel blockage and acute death of the mouse. Thus a further PEGylation procedure is indispensable.

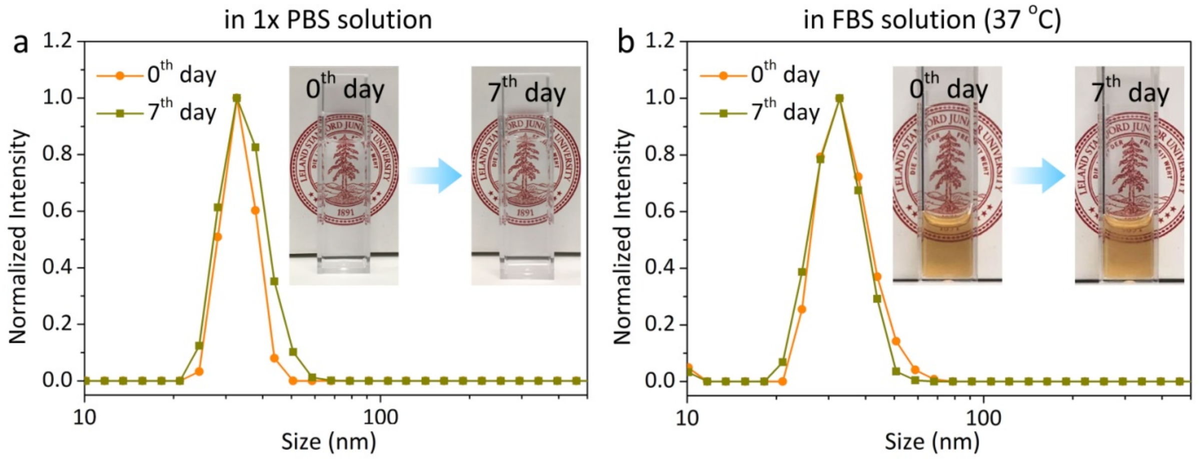

Supplementary Figure 5: Long-term stability of Er-RENPs@PMH-PEG in 1x PBS and FBS (37 ^{o}C) solution. DLS measurements of Er-RENPs@PMH-PEG in (a) 1x PBS solution and (b) 37 ^{o}C FBS at 0th and 7th day. The inset showed photographs of the clear solution at 0th and 7th day.

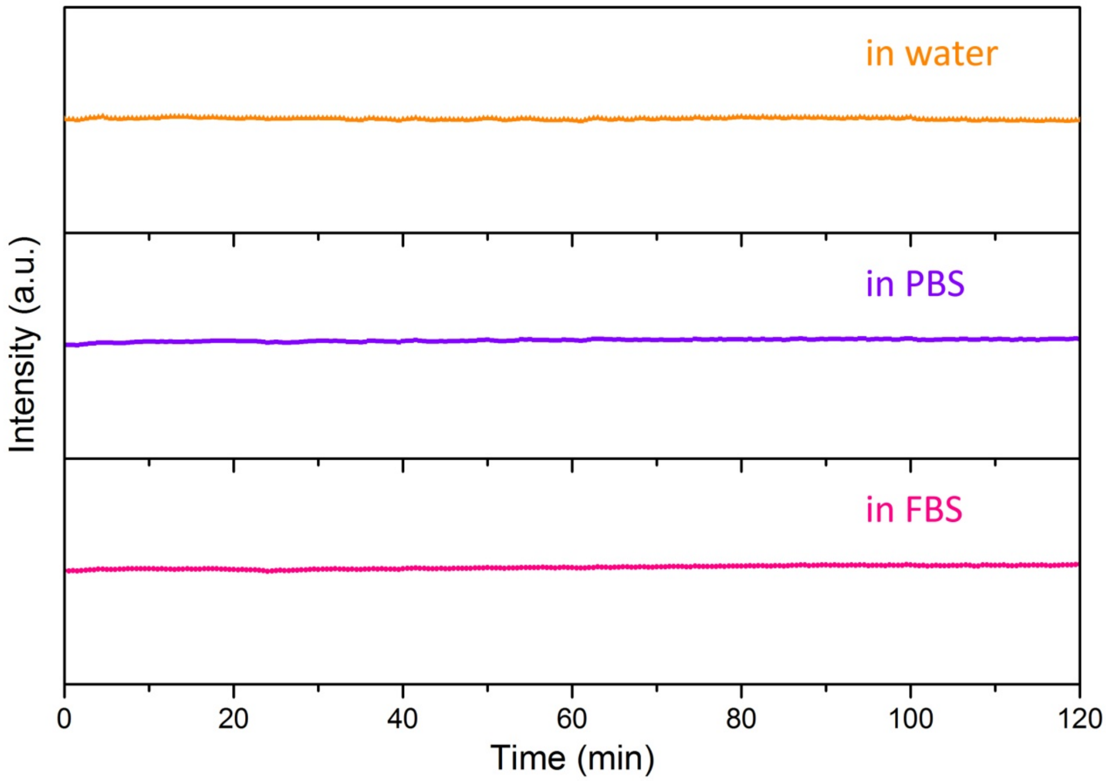

Supplementary Figure 6: Photostability of Er-RENPs@PMH-PEG in a variety of biological media. 1550 nm emission intensity of Er-RENPs@PMH-PEG in water, 1x PBS and fetal bovine serum (FBS) solution as a function of irradiation time excited by a 980 nm laser (power density: 150 mW·cm^{-2}).

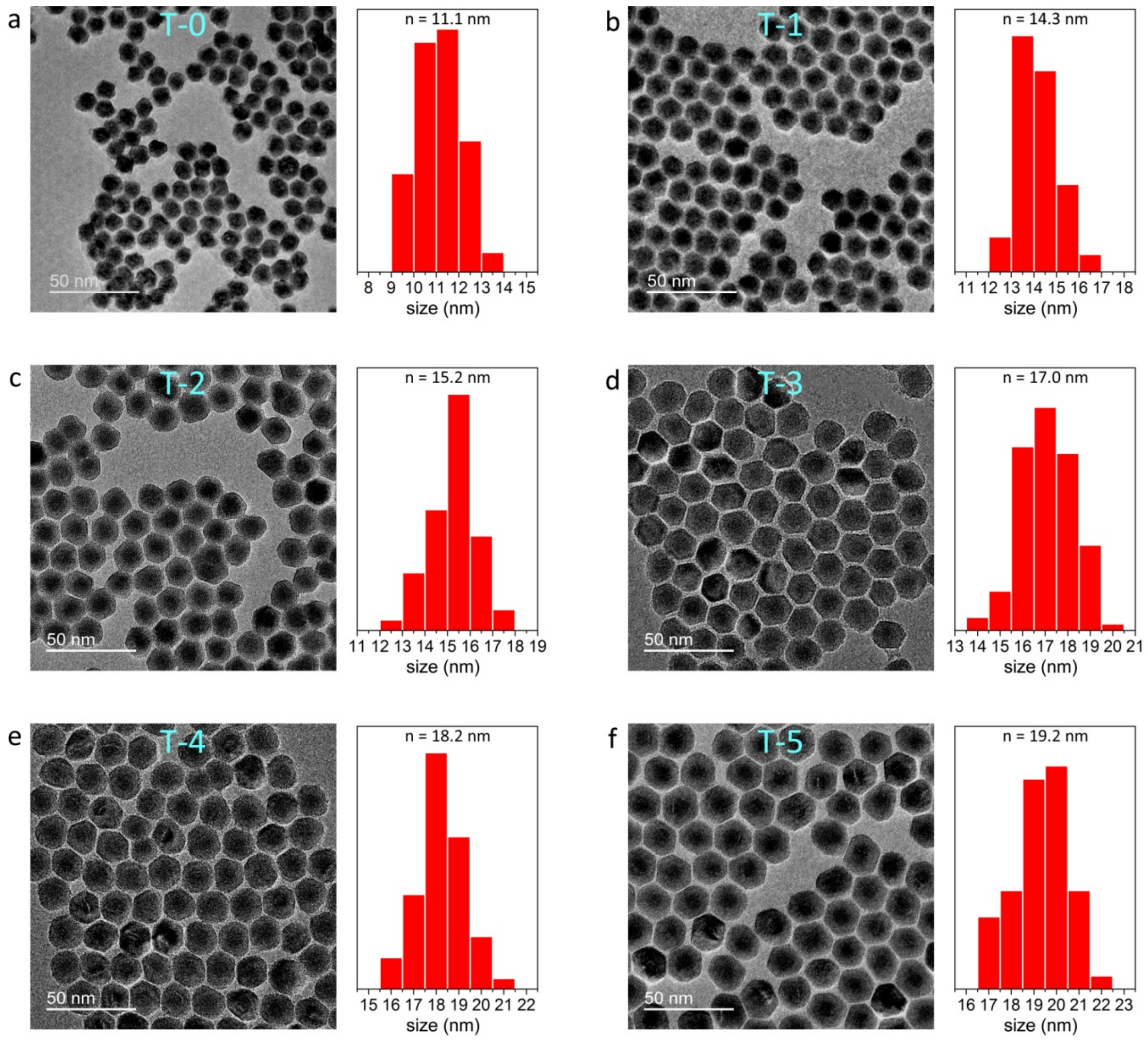

Supplementary Figure 7: Tuning the inert shell thickness of Er-RENPs. TEM images of the as-prepared $NaYbF_4$:2%Er,2%Ce@$NaYF_4$ nanoparticles with different $NaYF_4$ shell thickness. **a** showed the $NaYbF_4$:2%Er,2%Ce core nanoparticles with average size of 11.1 nm. Thus the shell thicknesses of $NaYbF_4$:2%Er,2%Ce@$NaYF_4$ nanoparticles in **b-f** were: (**b**) 3.2 nm, (**c**) 4.1 nm, (**d**) 5.9 nm, (**e**) 7.1 nm, (**f**) 8.1 nm, respectively.

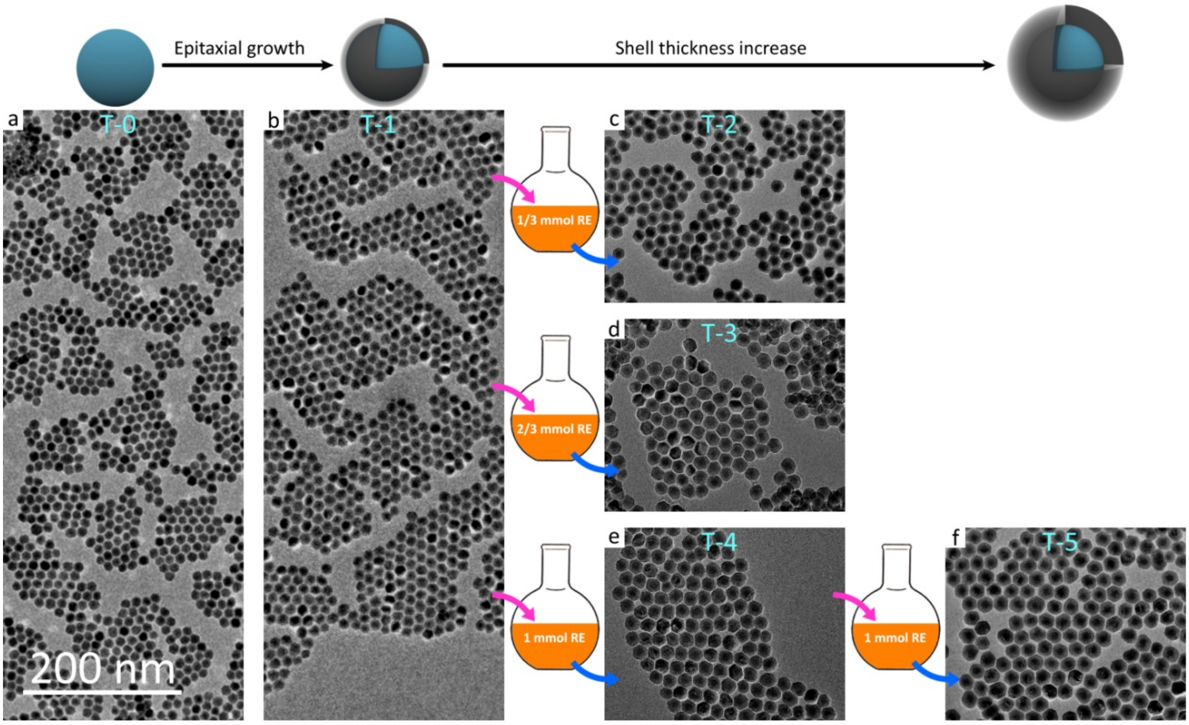

Supplementary Figure 8: Control experiments investigating shell growth of Er-RENPs. Our results showed that the inert shell thickness of Er-RENPs can be precisely controlled through a multi-step epitaxial seeded growth method. In a typical epitaxial growth procedure, the (**a**) $NaYbF_4$:2%Er,2%Ce core nanoparticles with average size of 11.1 nm acted as seeds for the growth of $NaYF_4$ inert shell. The first step (**b**) endowed the core particles with a shell of 3.2 nm by adding 1 mmol shell precursors. At the second step (**c**-**e**), by adding different amount of shell precursors, the shell thickness can be precisely controlled: (**c**) 4.1 nm, (**d**) 5.9 nm, (**e**) 7.1 nm. The shell thickness of Er-RENPs was further increased to (**f**) 8.1 nm by adding another 1 mmol shell precursors at the third step.

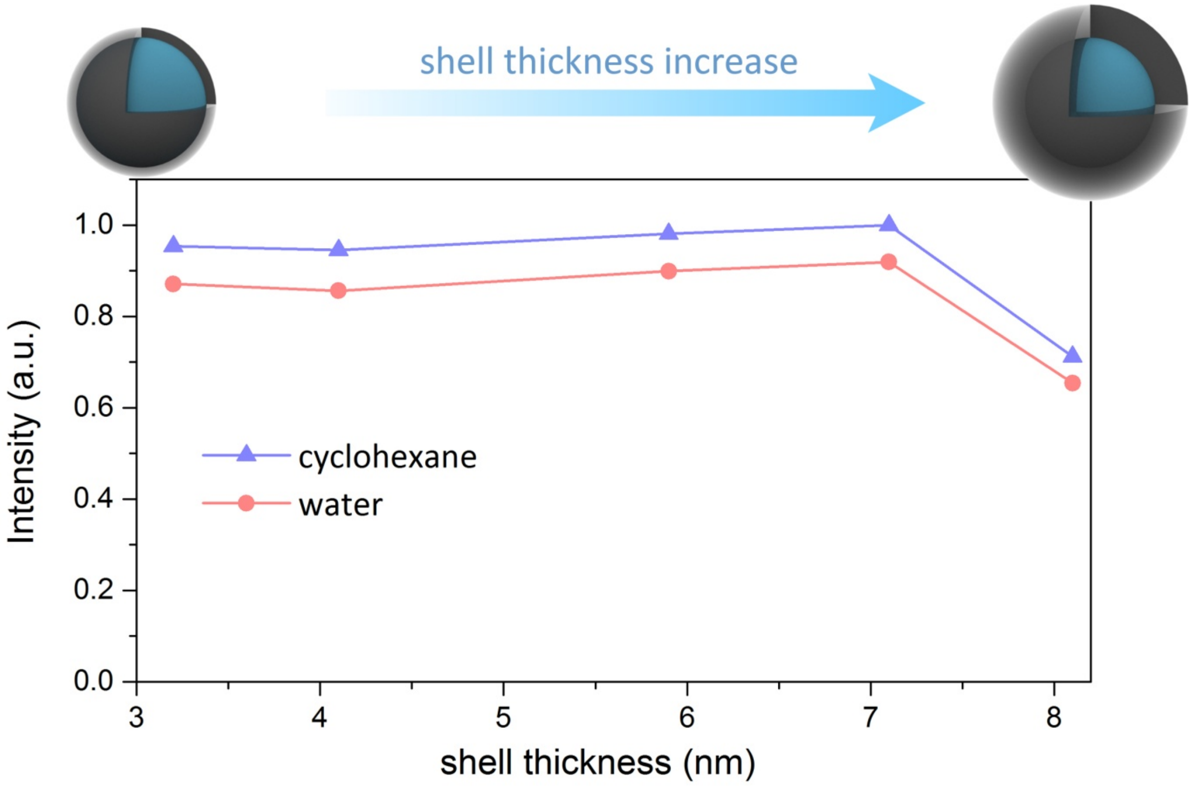

Supplementary Figure 9: Influence of shell thickness on the quenching effect of upconversion luminescence of Er-RENPs. The upconversion emission intensities of Er-RENPs in both cyclohexane and water phase were recorded as a function of shell thickness. Our results showed that even with a thin shell (3.2 nm), more than 90% of the upconversion luminescence of Er-RENPs retained after transferral from cyclohexane to water, indicating that the aqueous quenching effect to upconversion emission was much lower than the 1550 nm downconversion emission.

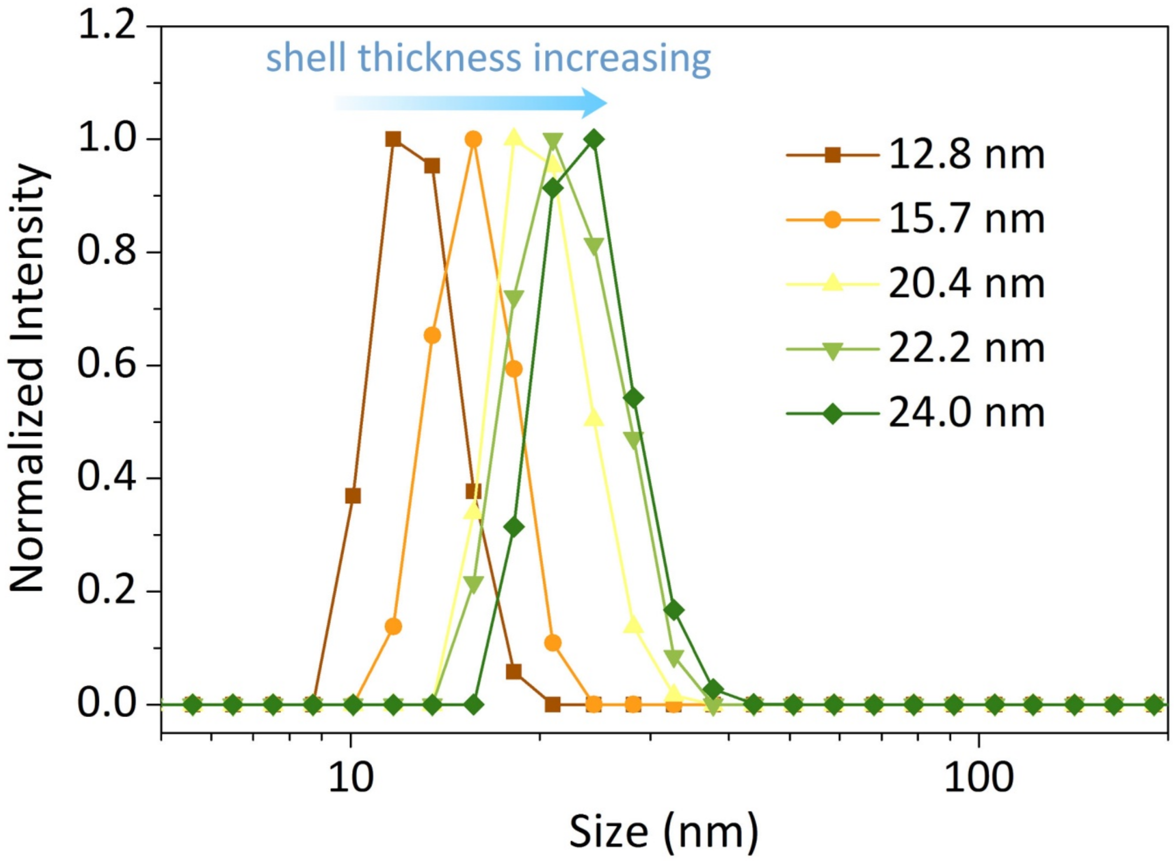

Supplementary Figure 10: DLS measurements of Er-RENPs with different shell thickness. Ligand-free RENPs were generated by the literature method[6] with slight modification. 1 mg RENPs dispersed in 50 μl cyclohexane were pumped into 1 ml ethanol containing 0.5 μl hydrochloric acid (36.5%) to form a clean solution. The solution was then centrifuged (15000rpm, 1hrs) to remove the excess hydrochloric acid and oleic acid. The supernate was discarded; and the RENPs were re-dispersed in 1ml ethanol to form a suspension of ligand-free RENPs for DLS measurement. The DLS data showed the increasing of shell thickness similar to the result of TEM data.

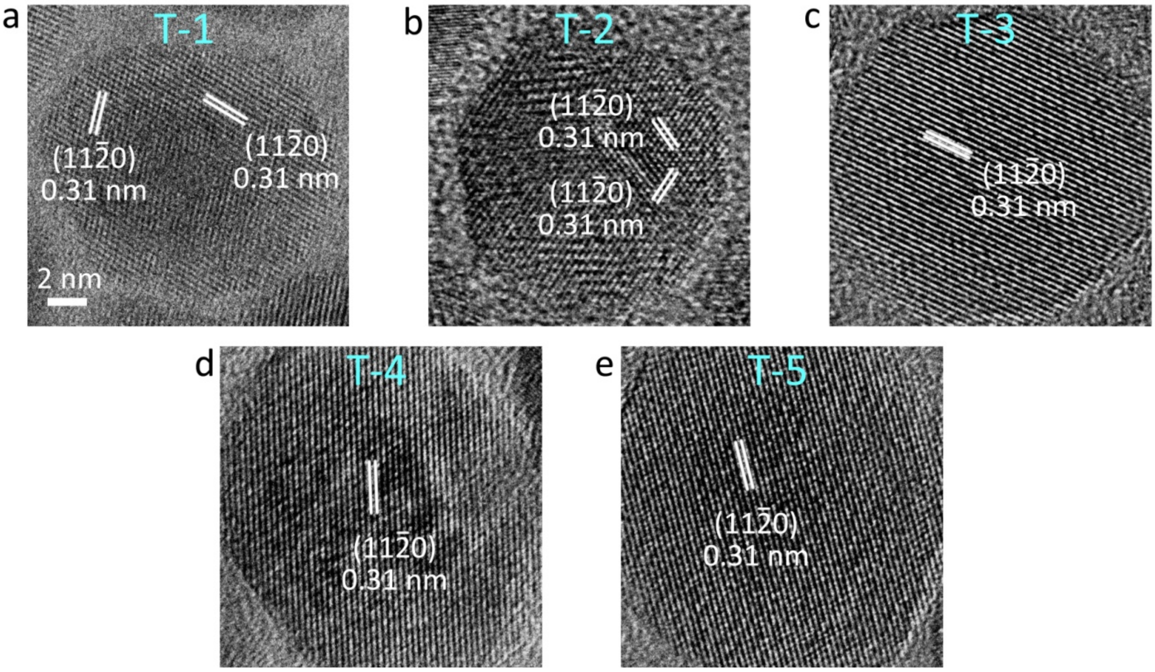

Supplementary Figure 11: High-resolution TEM images of Er-RENPs with different shell thickness. (**a-e**) High-resolution TEM images of Er-RENPs with different shell thickness revealed lattice fringes with similar spacing of about 0.31 nm indexed to the (110) planes of β phase of $NaYbF_4$. Note that the epitaxial seeded growth of rare-earth nanoparticles usually accompany with $\alpha \rightarrow \beta$ phase transition of the nanocrystals, which could dramatically influence the luminescence of rare-earth nanoparticles. Our results showed that no phase transition of the Er-RENPs occurred during epitaxial seeded growth. The enhanced NIR luminescence in water phase was mainly attributed to the reduced nonradiative energy transfer from the Er^{3+} ions in core to the surface OH^- groups.

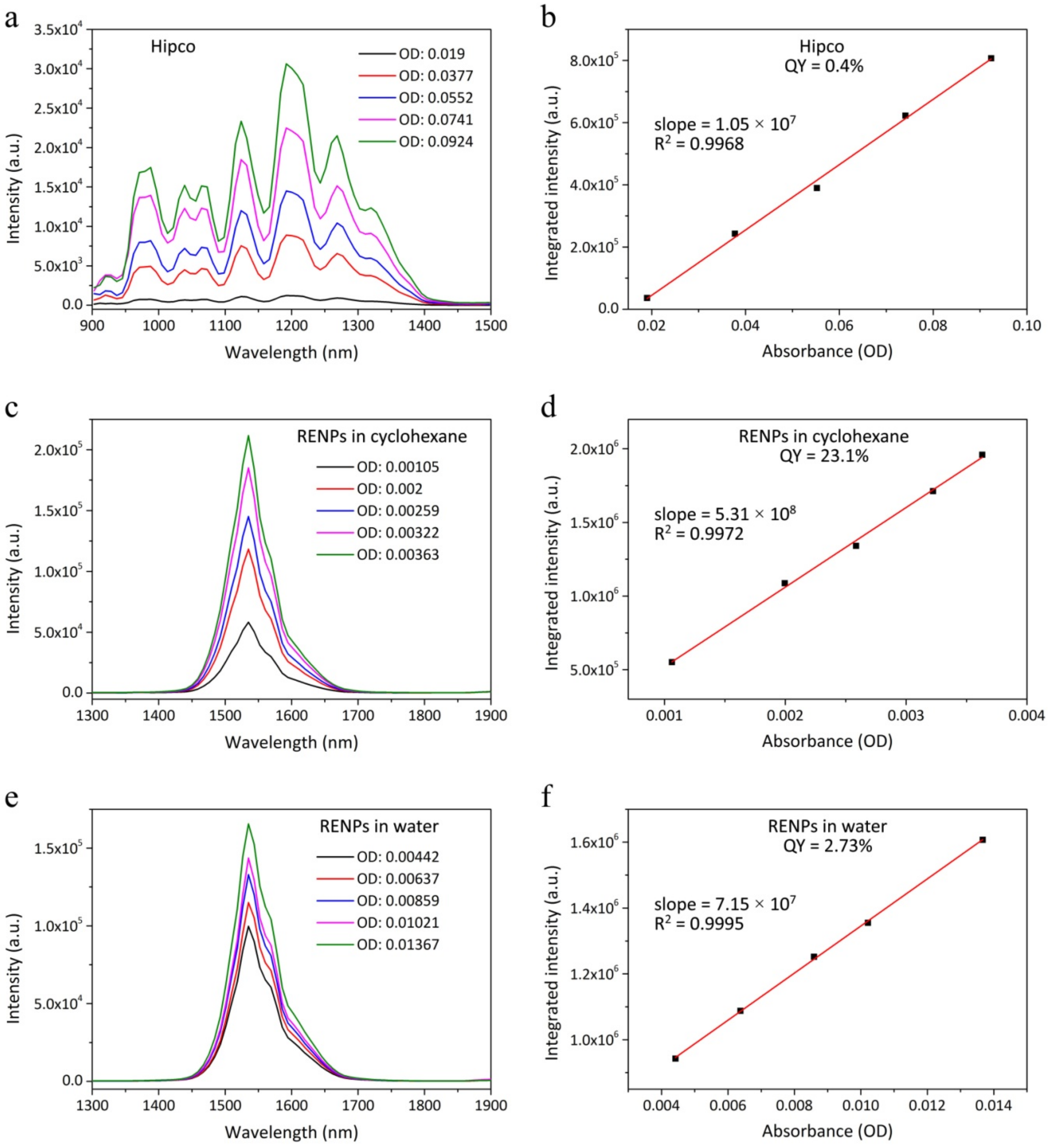

Supplementary Figure 12: Quantum yield measurements. (**a**) NIR fluorescence spectra of five Hipco solution in the range of 900-1500 nm under an excitation a 808 nm laser. (**b**) The integrated emission intensities of five fluorescence spectra of Hipco solutions plotted against the actual absorbance at 808 nm. The slope was obtained by linear fitting. (**c**) NIR luminescence spectra of five Er-RENPs in cyclohexane solution in the range of 1300-1900 nm under an excitation a 980 nm laser. (**d**) The integrated emission intensities of five luminescence spectra of Er-RENPs in cyclohexane solutions plotted against the actual

absorbance at 980 nm (QY = 23.1%). (**e**) NIR luminescence spectra of five Er-RENPs in aqueous solution in the range of 1300-1900 nm under an excitation a 980 nm laser. (**f**) The integrated emission intensities of five luminescence spectra of Er-RENPs in aqueous solutions plotted against the actual absorbance at 980 nm (QY = 2.73%).

The quantum yield of Er-RENPs was measured by using Hipco carbon nanotube (quantum yield of ~ 0.4%) as reference.[7] The Hipco was excited by 808 nm laser, while the Er-RENPs were excited by 980 nm laser using the same power density as the 808 nm laser (10 W·cm^{-2}). The quantum yield of Er-RENPs was estimated based on the following calculation[8]:

$$QY_{RENPs} = QY_{Hipco} \times \frac{Slope_{RENPs}}{Slope_{Hipco}} \times \left(\frac{n_{RENPs}}{n_{Hipco}} \right)^2 \tag{1}$$

Where QY_{RENPs} is the quantum yield of Er-RENPs, QY_{Hipco} is the quantum yield of Hipco carbon nanotube (~ 0.4%), n_{RENPs} and n_{Hipco} are the refractive indices of solvents (cyclohexane: 1.4209; water: 1.3290).

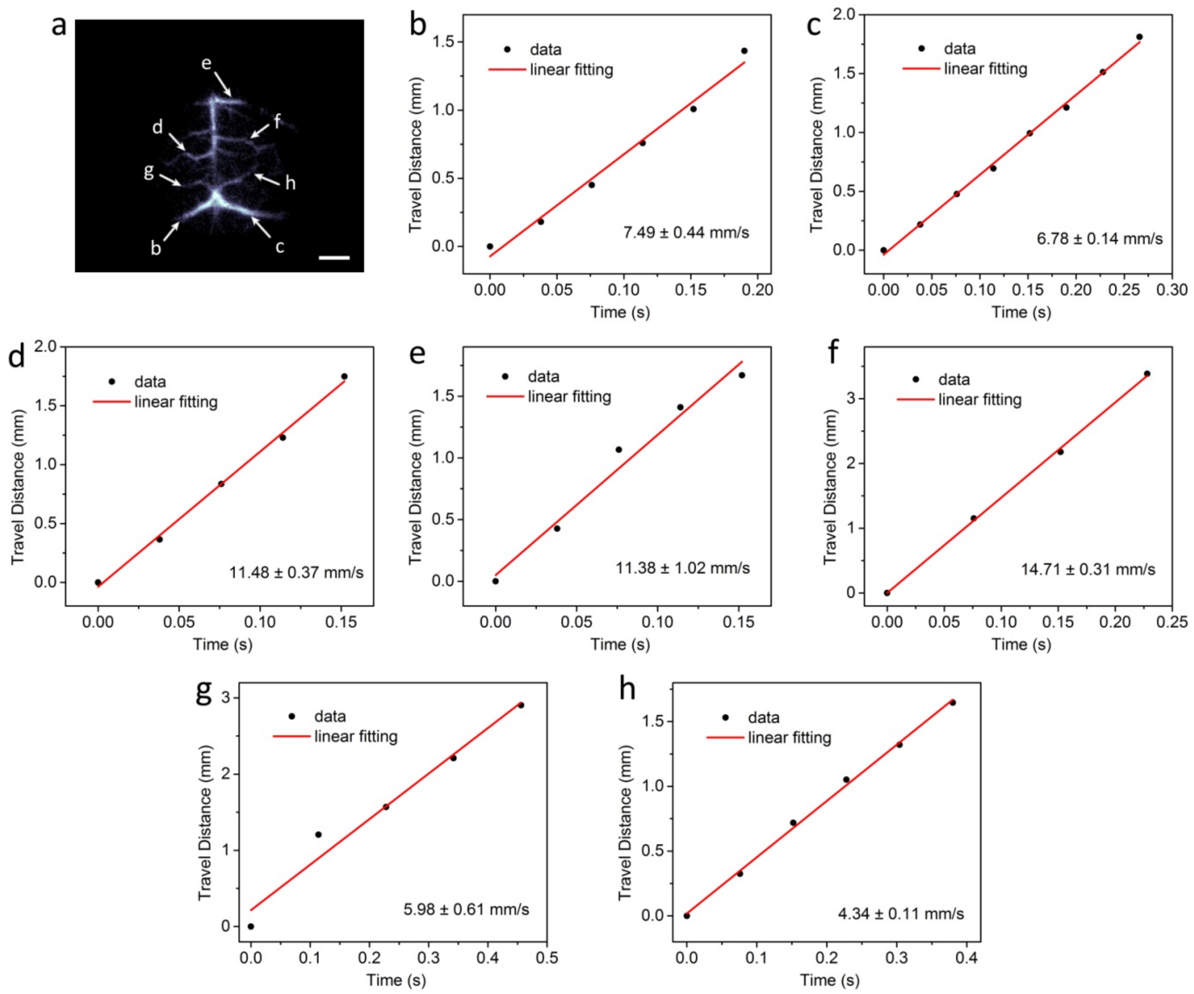

Supplementary Figure 13: Blood flow speed quantification in various cerebral vessels based on real-time NIR-IIb mouse brain imaging.[9] (**a**) A NIR-IIb image of mouse brain vessels after intravenously injecting a solution of Er-RENPs@PMH-PEG. The scale bar is 2 mm. (**b-h**) Blood flow velocity quantification by plotting the distance of blood front travelled against time. The linear fits revealed blood flow velocity in a range of approximately 4.34 to 14.71 mm·s^{-1}.

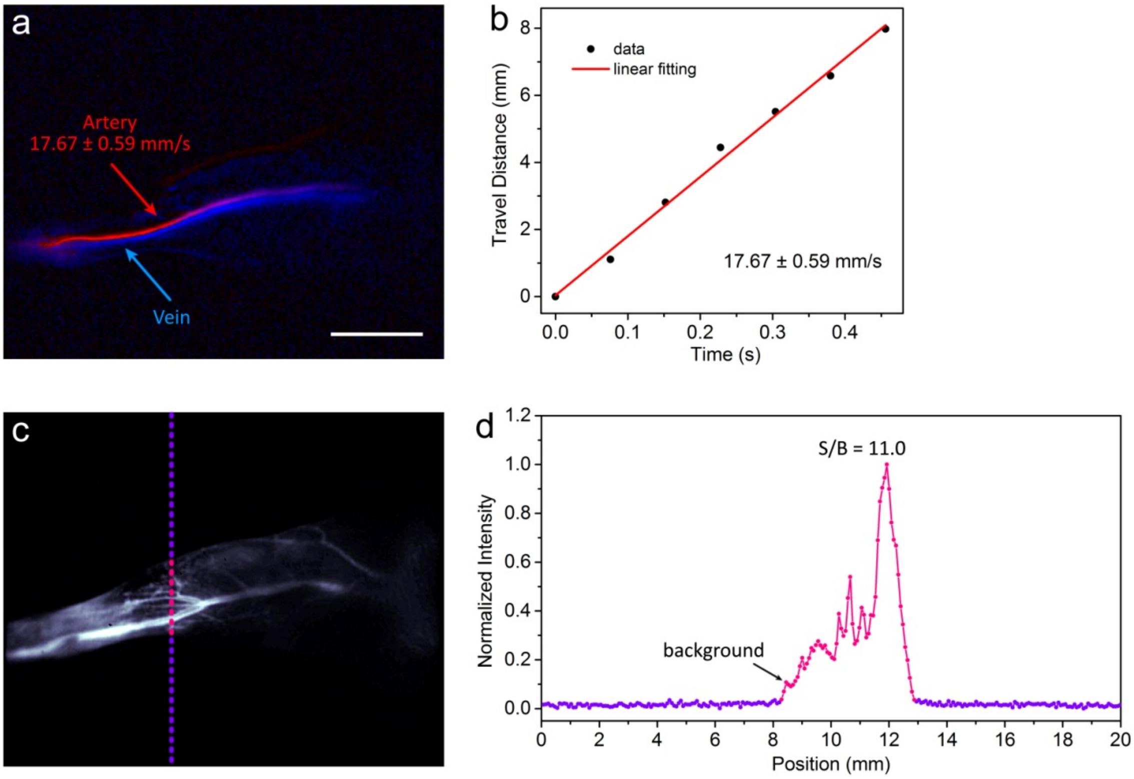

Supplementary Figure 14: Fast *in vivo* imaging of mouse hindlimb (Supplementary Movie 2). (**a**) PCA overlaid image showing the differentiation of arterial (red) and venous (blue) vessels. The scale bar is 4 mm. (**b**) A plot of the distance travelled by the arterial vessel as a function of time. The linear fit revealed a blood velocity of 17.67 $mm\cdot s^{-1}$. (**c**) NIR-IIb fluorescence image of the hindlimb of mouse (exposure time: 20 ms), with the corresponding signal-to-background ratio analysis shown in (**d**).

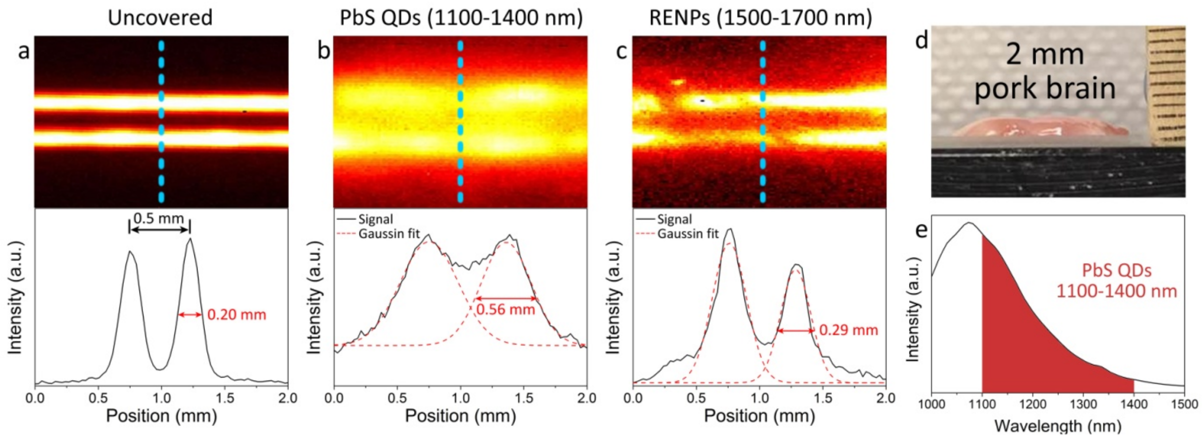

Supplementary Figure 15: Comparison of the NIR-IIa and NIR-IIb images. We performed a mock tissue phantom study by filling a pair of parallel capillary tubes with a mixture of 1100-1400 nm emitting PbS quantum dots (QDs) and our 1500-1700 nm emitting rare earth nanocrystals (RENPs). (a) The full width at half maximum (FWHM) of the tubes is 0.2 mm; the spacing is about 0.5 mm. These tubes were then immersed at depths of ~ 2 mm pig brain tissue excited by a 980 nm laser (150 mW·cm^{-2}), recorded in (b) 1100-1400 nm and (c) 1500-1700 nm emission windows, respectively. By measuring the Gaussian-fitted FWHM of the cross-sectional intensity profiles, the FWHM of the capillary tubes imaged in 1100-1400 nm and 1500-1700 nm region were found to be broadened by 180% (from 0.2 mm to 0.56 mm) and 45% (from 0.2 mm to 0.29 mm), respectively. (d) Photograph of the 2-mm pig brain tissue. (e) Emission spectrum of the PbS QDs. A 1100 nm long-pass filter and a 1400 nm short-pass filter were used to select the 1100-1400 nm emission region of the PbS QDs. A 1500 nm long-pass filter was used to select the NIR-IIb emission region (1500-1700 nm) of the RENPs.

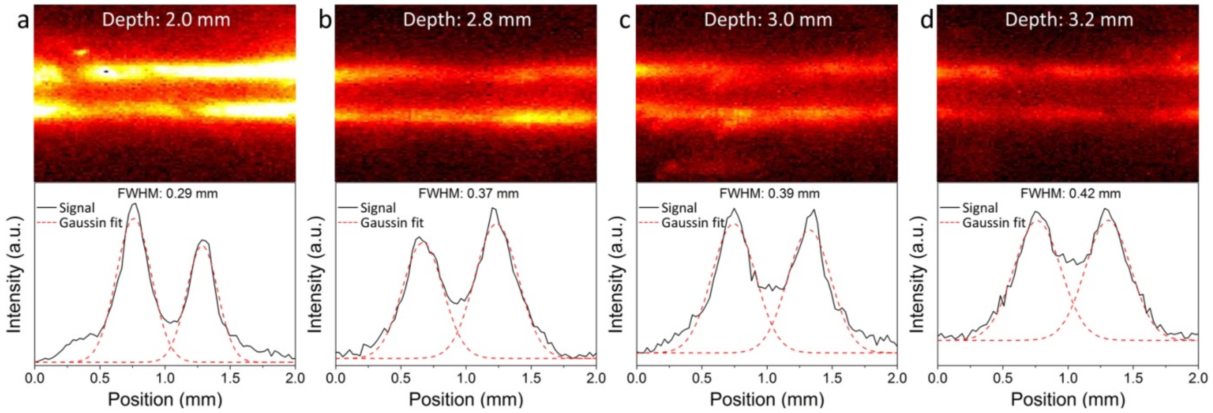

Supplementary Figure 16: Photon scattering phantom study using pig brain tissue. A pair of capillary tubes filled with RENPs (excited with 980 nm laser, 150 mW·cm^{-2}) were immersed under pig brain tissue with various thickness: (a) 2.0 mm, (b) 2.8 mm, (c) 3.0 mm, (d) 3.2 mm. By measuring the Gaussian-fitted FWHM of the cross-sectional intensity profiles, the apparent widths of the capillary tubes imaged in NIR-IIb region were found to be broadened by 110% (larger than 100%) at a depth of 3.2 mm.

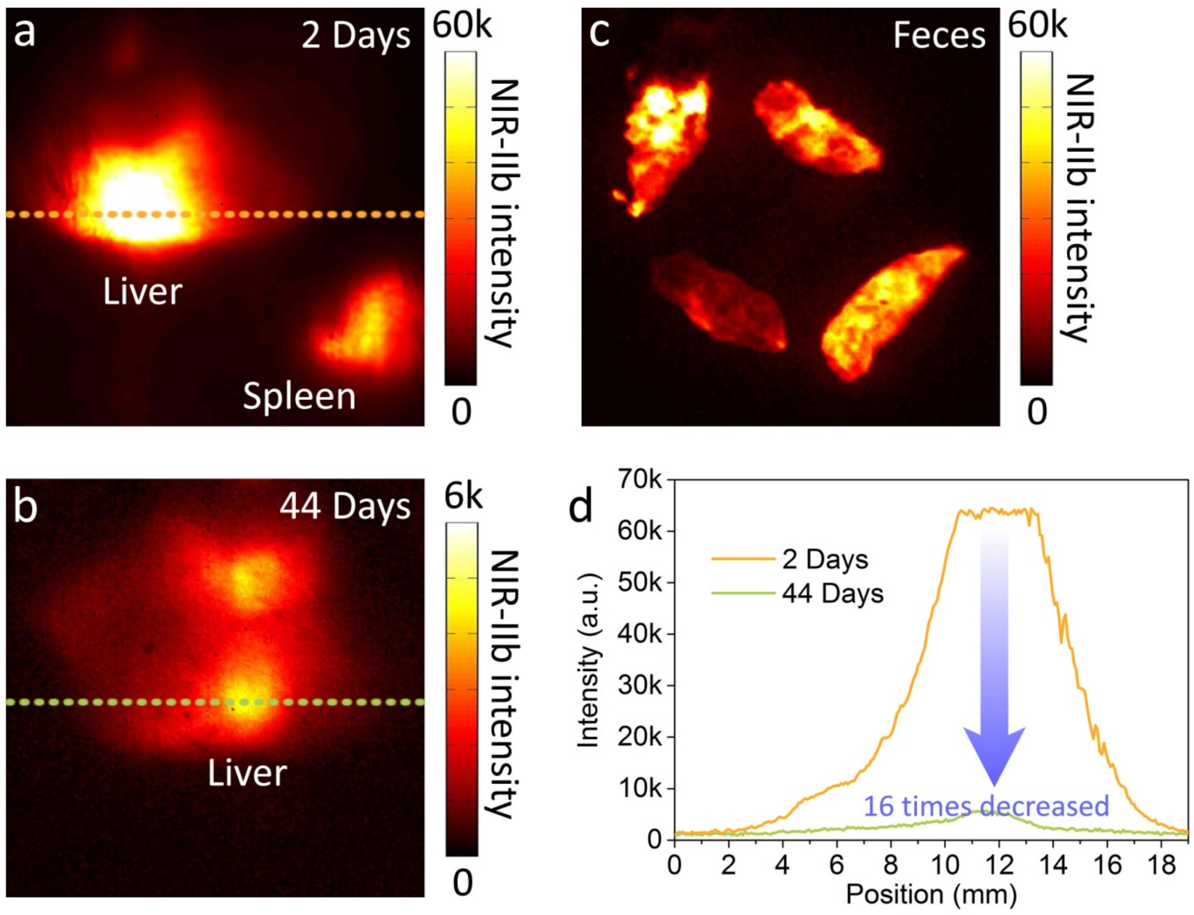

Supplementary Figure 17: Excretion of Er-RENPs@PMH-PEG. (**a**) A NIR-IIb fluorescence image of the liver of mouse captured at 2 days post injection. (**b**) A NIR-IIb fluorescence image of the feces of mouse collected at 2 days post injection. (**c**) A NIR-IIb fluorescence image of the liver of mouse captured at 44 days post injection. A semi-quantitative assessment of the excretion of the injected Er-RENPs@PMH-PEG was used by plotting the cross-sectional intensity profiles (**d**). Our result showed a dramatical intensity decrease of the liver signal at 44 days post injection to a low accumulation of only ~ 6%, suggesting high degree of excretion of the nanoparticles.

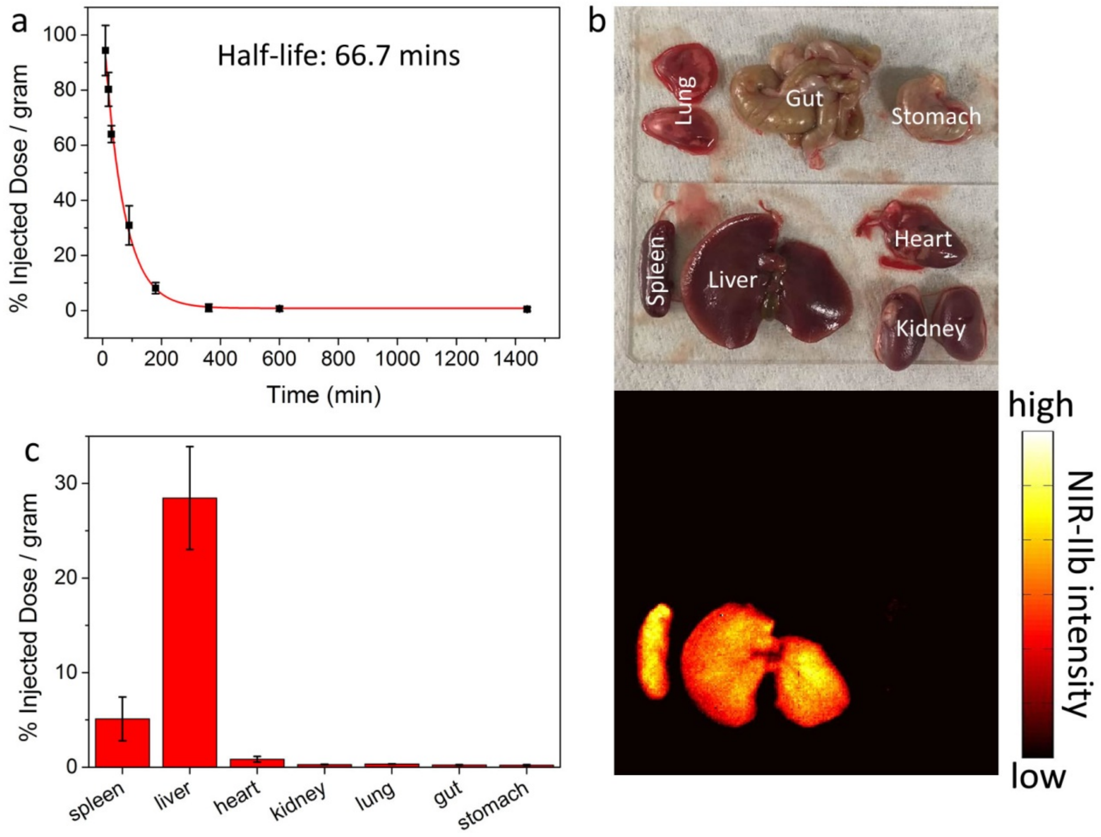

Supplementary Figure 18: Pharmacokinetics and bio-distribution of Er-RENPs@PMH-PEG. The clearance of Er-RENPs@PMH-PEG from mouse blood circulatory system was studied by intravenously injecting the RENPs to a group of C57Bl/6 mice (*n* = 2). Blood samples were collected and mixed with 1 mM EDTA solution at various time points post injection (up to 24 hours). (a) The half-life of the RENPs blood circulation was estimated to be 66.7 minutes, by measuring the concentration of the RENPs in the blood samples (based on the emission intensity). The bio-distribution of the RENPs in mice was also studied by sacrificing the mice at 24 hours post injection and collecting all the major organs. (b) Photograph (upper) and NIR-IIb image (lower) of the collected organs. The RENPs in each organ were fully extracted by dissolving the organ in 2% sodium cholate solution follow by sonication and heating at 60 ^{o}C for overnight. (c) The RENPs uptake in each organ collected from a group of C57Bl/6 mice (*n* = 2) was calculated based on the emission intensity of the RENPs in the organ extraction solution. The RENPs were found to

be largely accumulated in liver (28 percent injected dose per gram, or 28% ID/gram) and spleen (5% ID/gram) of the reticuloendothelial system (RES). While the uptakes in other organs were found to be lower than 1% ID/gram.

Supplementary References

1. Mai, H.-X. *et al.* High-quality sodium rare-earth fluoride nanocrystals: controlled synthesis and optical properties. *J. Am. Chem. Soc.* **128,** 6426-6436 (2006).
2. International Commission on Non-Ionizing Radiation Protection. Revision of guidelines on limits of exposure to laser radiation of wavelengths between 400 nm and 1.4 μm. *Health Phys.* **79,** 431-440 (2000).
3. Hong, G. *et al.* Through-skull fluorescence imaging of the brain in a new near-infrared window. *Nat. Photonics* **8,** 723-730 (2014).
4. Mai, H.-X., Zhang, Y.-W., Sun, L.-D., Yan, C.-H. Highly efficient multicolor up-conversion emissions and their mechanisms of monodisperse $NaYF_4$:Yb,Er Core and core/shell-structured nanocrystals. *J. Phys. Chem. C* **111,** 13721-13729 (2007).
5. Chen, G., Agren, H., Ohulchanskyy, T. Y., Prasad, P. N. Light upconverting core-shell nanostructures: nanophotonic control for emerging applications. *Chem. Soc. Rev.* **44,** 1680-1713 (2015).
6. Bogdan, N., Vetrone, F., Ozin, G. A., Capobianco, J. A. Synthesis of ligand-free colloidally stable water dispersible brightly luminescent lanthanide-doped upconverting nanoparticles. *Nano Lett.* **11,** 835-840 (2011).
7. Hong, G. *et al.* Ultrafast fluorescence imaging in vivo with conjugated polymer fluorophores in the second near-infrared window. *Nat. Commun.* **5,** 4206 (2014).
8. Diao, S. *et al.* Fluorescence imaging *in vivo* at wavelengths beyond 1500 nm. *Angew. Chem.* **127,** 14971-14975 (2015).
9. Hong, G. *et al.* Multifunctional *in vivo* vascular imaging using near-infrared II fluorescence. *Nat. Med.* **18,** 1841-1846 (2012).
