# Supplementary material for: Boosting the down-shifting luminescence of rare-earth nanocrystals for biological imaging beyond 1500 nm
Source: Nat Commun. 2017 Sep 29;8:737. doi: 10.1038/s41467-017-00917-6 (PMC5622117; doi:10.1038/s41467-017-00917-6)
Supplement: Supplementary file 2 — Description of Additional Supplementary Information [file 41467_2017_917_MOESM2_ESM.pdf]

Description of Additional Supplementary Files

File Name: Supplementary Movie 1
Description: Fast NIR-IIb in vivo dynamic imaging of Er-RENPs@PMH-PEG labeled cerebral blood flow in a C57Bl/6 mouse brain. Total video time: 41.8 s, frame rate: 25.6 frames/s.

File Name: Supplementary Movie 2
Description: Fast NIR-IIb in vivo dynamic imaging of Er-RENPs@PMH-PEG labeled blood flow in a C57Bl/6 mouse hindlimb. Total video time: 43.7 s, frame rate: 25.6 frames/s.
